# Effect of Gamma Radiation on the Chemical Structure and Physical Properties of Poly(butylene adipate-co-terephthalate)

**DOI:** 10.3390/polym18060683

**Published:** 2026-03-11

**Authors:** Daniel Marcos Rios, Mohammed Amine Atrous, Abderrahmane Belhaoues, Guillermina Burillo, Rodrigo Navarro, Ángel Marcos-Fernández

**Affiliations:** 1 Institute of Polymer Science and Technology (ICTP-CSIC), Juan de la Cierva, 3, 28006 Madrid, Spain; d.marcos@ictp.csic.es (D.M.R.); rnavarro@ictp.csic.es (R.N.); 2 Process Engineering Department, Faculty of Technology, 20 August 1955-Skikda University, Skikda 21000, Algeria; atrousmohammedamine@gmail.com (M.A.A.); ab.belhaoues@univ-skikda.dz (A.B.); 3 Instituto de Ciencias Nucleares, Universidad Nacional Autónoma de México, Circuito Exterior s/n, Ciudad Universitaria, Delegación Coyoacán, Mexico City C.P. 04510, Mexico; burillo@nucleares.unam.mx

**Keywords:** PBAT, gamma, chemical changes, chain scission, crosslinking

## Abstract

This study presents the effect of gamma rays of up to 2000 kGy on the chemical structure and the physical properties of a poly(butylene adipate-co-terephthalate) (PBAT) with 48% mol of terephthalic units. PBAT is a polymer with properties similar to polyethylene (PE) but it is biodegradable and not toxic to the environment, and it can be prepared with a renewable content of up to 68.6% weight, with uses in biomedicine and packaging. Previous studies found in the literature have been conducted using low doses and the results were contradictory. The results for gel content and crosslinking efficiency were in agreement with the results found in the literature. Molecular weight decreased and widened with the increase in dose. Proton NMR analysis was used for the first time in PBAT to determine the changes in chemical species, the formation of new chemical species, and the bonds more susceptible to be broken by gamma rays. Both thermal and mechanical properties were explained by the scission of the chains in the amorphous phase and at the boundaries of the crystallites. The thermal parameters most affected by irradiation were the crystallization temperature and temperature of melting after cooling from the melt. Stress and strain at break suffered a continuous decrease with dose until PBAT became fragile at high dose.

## 1. Introduction

Poly(butylene–adipate-co-terephthalate) (PBAT), sold under several brand names including Ecoflex^®^, Ecovio^®^, and Origo-Bi^®^, is a biodegradable aliphatic–aromatic copolyester composed of randomly distributed repeating units derived from 1,4-butanediol, adipic acid, and terephthalic acid. The polymer backbone consists of two primary segments: the aliphatic butylene adipate (BA) units and the aromatic butylene–terephthalate (BT) units. The introduction of a co-monomer in the PBT chemical structure modifies and tunes PBAT properties such as its thermal and mechanical behaviour or biodegradability rate. The aliphatic segments provide flexibility, softness, and biodegradability, while the aromatic segments contribute to rigidity, mechanical strength, and thermal stability thanks to the benzene rings present in terephthalate units [1].

PBAT is considered a petroleum-based polymer [2], although adipic acid and 1,4-butanediol can be obtained by fermentation and therefore up to 68.6% of the weight of the polymer can be of sustainable origin. The production of bio-based PBAT might greatly lessen its negative effects on the environment and bring it closer to the objectives of the circular economy.

Growing environmental prohibitions on single-use plastics and the demand for biodegradable substitutes that retain practical qualities similar to polyethylene have drawn a lot of interest to PBAT. PBAT has been considered as a viable alternative for low-density polyethylene (LDPE) due to their similar mechanical properties. The glass transition temperature of PBAT is well below room temperature; therefore, it possesses a broad workability range which allows it to be processed through extrusion, injection moulding and thermoforming. Its commercialisation has been mainly devoted to biodegradable packaging [3], but the use of these polymers in the biomedical field has recently attracted considerable attention because of their biodegradability and lack of toxicity [4,5].

PBAT does not degrade in pure marine and fresh water [6], but under appropriate conditions, microorganisms [7] and enzymes can attack PBAT due to the hydrolyzable butylene–adipate ester bonds in the polymer backbone. PBAT is fully biodegradable under industrial composting conditions [8] as well as in soil [9].

The EN 13432 standard [10] in Europe, which calls for at least 90% biodegradation and disintegration within six months under industrial composting settings, and the US standard ASTM D6400 [11] are met by PBAT. Furthermore, TÜV Austria has certified PBAT as “OK Compost Industrial” and “OK Biodegradable Soil,” confirming its usage in agricultural and compostable packaging applications [12].

The molecular weight, manufacturing parameters, and degree of crystallinity all affect the tensile strengths of PBAT, which has a high elongation at break, which frequently exceeds 400–700%, surpassing most biodegradable polyesters such as PBS and PLA [3].

In both applications, packaging and biomedical, PBAT is susceptible to be sterilized with, among other methods, ionizing radiation (e-beam and gamma), which has an advantage over steam and heat sterilization methods (at temperatures too high for PBAT to maintain its dimensional stability) in that it can be carried out at ambient temperature, and over ethylene oxide in that no residual chemicals are left in the material [13]. Ionizing radiation can be considered a green technology with very low VOC emissions and no solvent waste. Furthermore, gamma radiation is often preferred over electron beam (e-beam) in industrial applications due to its greater penetration depth, allowing the uniform treatment of bulk materials or densely packed products. In addition to sterilization, gamma radiation is a method for tuning the physicochemical properties of polymers, and some polymers can upgrade their properties via radiation, changing from commodity polymers to engineering polymers and from engineering polymers to high performance polymers [14].

When a polymer is subjected to gamma irradiation, the energy is absorbed and transferred to the electrons in the material, leading to the ionization and excitation of atoms and molecules. This primary interaction results in the formation of free radicals, which are highly reactive and initiate a series of secondary reactions within the polymer matrix. The overall effect of gamma radiation on a polymer depends on several variables, including the polymer’s chemical structure, molecular weight, degree of crystallinity, presence of additives or oxygen, and the irradiation conditions such as dose, dose rate, and atmosphere [15,16,17].

Gamma radiation can produce two main events on a polymeric chain, chain scission and crosslinking. Chain scission leads to a decrease in molecular weight and therefore a deterioration of the polymer’s mechanical properties. Crosslinking, with the ultimate formation of a three-dimensional network, significantly alters the material’s mechanical strength, solvent resistance, stiffness, thermal properties and thermal stability.

Since the commercial introduction of PBAT in 1998 under the brand name Ecoflex^®^ [18], the effect of ionizing radiation on PBAT has been scarcely studied. Only twenty works involving PBAT irradiation can be found, seven of them for gamma irradiation [19,20,21,22,23,24,25] and thirteen of them for electron beam irradiation [26,27,28,29,30,31,32,33,34,35,36,37,38]; more than half of these works were published after the year 2020. Both ionizing radiations will be considered as having the same effect [39].

Most of the works studied blends of PBAT with other polymers, such as PCL [33,35], PLA [23,25,26,31,32,34,38], PGA [38], starch [21], and PBS [20], or with additives such as multi-walled nanotubes [37], triallylisocyanurate (TAIC) [29], orange essential oil [22,24], and silsesquioxane [28], and only three of them are focused on pure PBAT [19,27,30] (as it will be seen later, one of these works [27] is not pure PBAT, although some of the works with blends and additives studied the pure PBAT as reference [22,24,26,28,29,31,32]).

The maximum dose irradiated to PBAT in these works was 300 kGy [19], with two works reaching 200 kGy [26,28] and the rest working with doses of 100 kGy [30,32] or lower [22,24,29,31].

When the results for PBAT irradiation in these works are analyzed, no defined trend on the molecular weight or the physical properties can be drawn from the literature’s data. Some authors find no significant change in molecular weight at doses up to 100 kGy [31,32], whilst others find a slight decrease followed by a significant decrease (up to 50 kGy) [24] or an increase up to 90 kGy [29]. For the melting enthalpy in the second heating cycle, the results are contradictory, and in one work it increased very significantly (up to 50 kGy) [24], and in another work it decreased significantly (up to 300 kGy) [19]. For the tensile strength, up to 30 kGy, in some cases there is no significant change [24,29,31,32] and in other cases there is a slight increase [19,26], and above 50 kGy to 300 kGy, most of the data show a tendency to decrease. When strain at break is evaluated, the results are even more confusing than for tensile strength. No significant change is found in some works [26,29,31,32] with doses up to 200 kGy, though a slight increase is found in another work up to 25 kGy followed by a slight decrease at 50 kGy [24], and a very significant and continuous decay is found in another work [19]. Thus, it is completely unclear the effect of irradiation up to 300 kGy on the strain at break. For Young’s Modulus results, all works do not observe a significant effect on this parameter [19,24,31,32] except for one, where a continuous decrease is found [29].

Leaving aside the difference in absolute values, which could be due to the differences in the monomers in the copolymer (ratio adipate/terephthalate), not known in many of these works, and differences in the experimental conditions of the analysis, it can be seen that results in the literature sometimes are not consistent. Furthermore, the doses evaluated in these works are relatively low and may be not enough to establish clear relationships between the dose and its effect on the physical properties. In addition, no study has addressed the effect of ionizing irradiation on the chemical structure of PBAT

We present a detailed study on the effect of the irradiation of PBAT within a wide range of doses, up to 2000 kGy, in order to establish clear relationships for the thermal properties and to determine the detailed effect on the mechanical properties. Furthermore, by NMR, we have studied the stability of the different bonds in the PBAT, and proposed a mechanism for the chain scission and the crosslinking of the PBAT polymer chains.

By integrating these insights with comprehensive data on gel content, molecular weight evolution, thermal transitions, and mechanical performance, we offer a holistic understanding of how gamma radiation tailors the structure and properties of PBAT, which represents a contribution to the current state of the art and provides a fundamental framework for predicting the behaviour of this polymer when subjected to high-energy radiation.

## 2. Experimental

### 2.1. Materials

Poly(butylene adipate-co-terephthalate) (PBAT) in the form of white pellets was provided by NaturePlast (Mondeville, France) with the reference name PBE 006. This grade is intended for extrusion applications and exhibits a Melt Flow Index (190 °C, 2.16 kg) of 4–6 g·10 min^−1^, a melting temperature of 110–115 °C, a tensile strength at break greater than 17 MPa, and a tensile elongation at break exceeding 590%.

### 2.2. Sample Preparation

PBAT was processed into a thin sheet by extrusion using a Xplore MC 15 HT micro compounder, with a cast die and a micro casting line (Xplore Instruments BV, Sittard, The Netherlands). PBAT pellets were loaded in the microcompounder and extruded at 160 °C and 40 rpm. A sheet was cast from the PBAT that was melted exiting a flat die, using a three-roll micro-casting line with, first, a speed-controlled calendar that defined the thickness of the sheet, second, a transport roll with an additional pinch roller to enable grip adjustment on the sheet, and third, a torque-controlled winder roll for a constant and reproducible winding of the sheet.

A continuous thin sheet, 5 cm wide and approximately 450 microns thick, was obtained, from with test specimens were cut (Figure S1 in the Supplementary Materials).

### 2.3. Gamma Irradiation

Pieces of approximately 0.25–0.35 g for gel content measurements and type 5B dumbbell test pieces (according to ISO 527-2 [40]) for thermal and mechanical properties were cut from the PBAT sheet. The samples were introduced into 9 cm × 6 cm polyethylene bags and sealed in air. The samples were irradiated in air at room temperature with a ^60^Co–source (Gammabeam 651-PT, Nordion International Inc., Ottawa, ON, Canada) installed at the Instituto de Ciencias Nucleares of UNAM, México, with an activity of 63,000 Ci at a dose rate of 10.07 kGy h^−1^ and doses from 200 to 2000 (200, 500, 700, 1200, 1500 and 2000) kGy for dumbbell pieces and 10 to 2000 (10, 20, 30, 50, 100, 200, 500, 700, 1000, 1200, 1500, and 2000) kGy for gel content pieces. The determination of the dose rate for the gamma ray source was carried out with modified Fricke (ferrous sulfate dosimeter modified by the addition of cupric sulfate) [41]. The dosimetric standard for dose rate was prepared on 17 August 2024. Once the absorbed dose was determined by the Fricke dosimeter, the absorbed dose in the polymer, referred as dose in this work, was calculated.

### 2.4. Gel Content

To evaluate the gel content, 230–330 mg of gamma-irradiated samples was immersed in 10 mL chloroform for 5 h at room temperature. Solvent was decanted and 10 mL of fresh chloroform was added. After 24 h at room temperature, the decantation and addition of fresh solvent was repeated and left for further 24 h. Finally, chloroform was decanted, and the resulting gel was dried in air for 2 h and in vacuum at room temperature overnight. The remaining dry solid was weighed. The gel content was calculated from the weight ratio of the insoluble fraction after irradiation and the sample prior to irradiation.

$$Gel\;content\;\left(\%\right)=\frac{w_{e}}{w_{i}}\times 100$$

where w_e_ is the weight after extraction with chloroform and w_i_ is the initial weight before extraction.

Two samples were measured for each dose in order to check the dispersion of the data.

### 2.5. Characterization Methods

Size Exclusion Chromatography (SEC). A Waters 1515 gel permeation chromatograph equipped with an isocratic 1515 HPLC pump and refractive index detector Waters IR2414 was used for molecular weight determination (Waters Corporation, Milford, MA, USA). A set of HR4, HR1 and HR0.5 Waters columns conditioned at 35 °C was used to elute samples at 1 mL·min^−1^ flow rate with HPLC-grade THF as solvent. Polystyrene standards (2970–200,000 g·mol^−1^) were used for the calibration. Standards above 200,000 g·mol^−1^ showed that above this value the limit of total exclusion of the columns was reached.

The samples were dissolved (approximately 5 mg in 1 mL) in HPLC-grade THF and filtered through a 0.45 μm PTFE filter to eliminate any particles that could clog the columns.

Nuclear Magnetic Resonance (NMR). Solution proton NMR spectra were recorded at room temperature in a Bruker Avance III HD instrument (Bruker BioSpin, Billerica, MA, USA) working at 400 MHz, using deuterated chloroform (CDCl_3_) as the solvent. Spectra were referenced to the residual solvent signal at 7.26 ppm. Approximately 10 mg of the polymer was dissolved in 0.8 mL deuterated solvent.

Differential Scanning Calorimetry (DSC). The thermal transitions of the samples were analyzed by DSC on a Mettler Toledo DSC 822e calorimeter (Mettler Toledo, Schwerzenbach, Switzerland) equipped with a liquid nitrogen accessory. Discs cut from tested tensile specimens and from specimens for gel content measurement, weighing approximately 15 mg, were sealed in aluminum pans with perforated lid. The samples were heated, from 25 °C to 180 °C at a rate of 10 °C·min^−1^, cooled at −80 °C at a rate of −10 °C·min^−1^, maintained for 5 min at this temperature and re-heated from −80 °C to 180 °C at a rate of 10 °C·min^−1^. All scans were carried out under a constant nitrogen purge of 30 mL·min^−1^. Melting points (T_m_) and crystallization temperatures (T_c_) were taken as the maximum of the endothermic transition and the minimum of the exothermic transition respectively, whereas glass transition temperatures (T_g_) were taken as the midpoint of the change in heat capacity. The crystallinity of the samples was calculated by taking the enthalpy of fusion of the 100% crystalline PBT homopolymer (ΔH_0_) as 32.0 kJ·mol^−1^ (145 J g^−1^) [42] as shown in the following equation.

$$Crystallinity\;\left(\%\right)=\frac{{∆H}_{m}}{{∆H}_{0}\;w}\times 100$$

where ΔH_m_ is the melting enthalpy (J·g^−1^), (or the crystallization enthalpy, ΔH_c_) measured as the area under the melting (or crystallization) peak during the DSC heating (or cooling) scan, ΔH_0_ is the melting enthalpy for 100% crystalline PBT homopolymer (145 J·g^−1^) and w is the weight fraction of PBT in the sample (w = 0.5 for the PBAT of this study).

Temperature and enthalpy were calibrated with the Indium standard (T_m_ (onset) = 156.60 °C; ΔH_m_ = 28.45 J·g^−1^). At least two samples were measured for each dose in order to check the dispersion of the data.

Mechanical properties. Tensile properties were measured at ambient temperatures in an Instron testing machine model 4301 (Instron, Norwood, MA, USA) equipped with a 1 kN load cell. Type 5B dumbbell test pieces (according to ISO 527-2 for tensile testing of plastics; same test specimen as Type 4 dumbbell according to ISO 37 [43] for rubber) were cut from the extruded sheets. A crosshead speed of 5 mm·min^−1^ was used. The strain was measured using the crosshead separation and referred to 10 mm of initial length.

A minimum of five samples were tested for each material. The calculation of the error in the measurements was performed using a *t*-test.

## 3. Results and Discussion

### 3.1. PBAT Characterization

Prior to irradiation, the unirradiated material was characterized for its chemical structure, thermal properties and mechanical properties.

**Figure 1:**
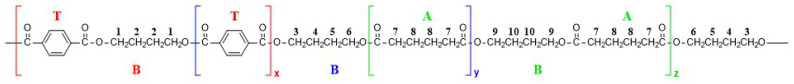
Chemical structure of PBAT. T = terephthalic ring; B = butylene units; A = adipate unit.

In Figure S2 in the Supplementary Materials, the proton NMR spectrum of the unirradiated material is shown. In Figure 1, the chemical structure with the labelling of the protons according to the literature [44] is presented.

A singlet at 8.09 ppm corresponds to the four protons of the terephthalic ring (T).

The signals in the interval 4–4.5 ppm are due to butylene (B) units. The signals correspond to two pseudo-singlets and two triplets. The pseudo-singlet at 4.43 ppm corresponds to methylene units linked to the oxygen when butylene is in between two terephthalate units (1); the triplet at 4.37 ppm corresponds to methylene units linked to the oxygen of terephthalate unit when butylene is in between one terephthalate unit and one adipate unit (A) (3); the triplet at 4.14 ppm corresponds to methylene units linked to the oxygen of adipate unit when butylene is in between one terephthalate unit and one adipate unit (6); and the pseudo-singlet at 4.08 ppm corresponds to the methylene units linked to the oxygen of adipate unit when butylene is in between two adipate units (9).

The pseudo-singlet at 2.32 ppm corresponds to the methylene units linked to the carbonyl of adipate units (7).

The pseudo-singlet at 1.97 ppm corresponds to the internal methylene units of butylene when in between two terephthalate units (2).

The multiplets at 1.84 and 1.81 ppm correspond to the internal methylene units of butylene when in between one terephthalate unit and one adipate unit (4,5).

The pseudo-singlet at 1.68 ppm, merged with the pseudo-singlet at 1.65 ppm, correspond to the internal methylene units of butylene when in between two adipate units (10), and to the internal methylene units of the adipate units, respectively (8).

From the areas of the integral at 8.09 ppm (T) and 2.32 ppm (7, A), a composition of 52% mol adipate units and 48% mol of terephthalate units in the copolymer is calculated; thus, they are almost equimolecular.

From the areas of the peaks at 4.43 (1), 4.37 (3), 4.14 (6) and 4.08 (9) ppm, the ratios of the butylene units in between terephthalic units (1, T-B-T), in between one terephthalate unit and one adipate unit (3,6, T-B-A and A-B-T) and in between adipate units (9, A-B-A) were calculated: 16.4%, 65.8% and 17.8% mol, respectively.

Following the method reported in the literature [44], the block lengths of terephthalate units (BT) and adipate units (BA) were calculated, with values of 1.46 and 1.58 respectively; thus, they are very short sequences, and the degree of randomness had a value of 1.31, which means that the copolymer is mainly random.

From the spectrum it can be concluded that PBAT is a random copolymer that has almost a 50:50 mol ratio of terephthalate: adipate (48:52 actually), and that more than half (65.8% mol) of the butylene units are in between the terephthalate and adipate units, which will not be able to crystallize, limiting the crystallization ability of the copolymer, which could have very short pure butylene–terephthalate (BT) and pure butylene–adipate (BA) sequences that could form crystals in a phase-separated phase.

From thermogravimetric analysis it was found that all the material degraded without any significant amounts of residue; thus, the PBAT copolymer is a pure polymeric material without fillers. Decomposition started at temperatures above 300 °C; thus, at the processing temperature, the material is thermally stable.

SEC analysis provided a number average molecular weight (M_n_) of 46,400 Da, and a weight average molecular weight (M_w_) of 104,000 Da with a polydispersity index (PI) of 2.2. The PI is wide, as expected for a polycondensation polymer, and the high molecular weight assures its good mechanical properties.

In Figure S3 in the Supplementary Materials, the calorimetric curves for PBAT are shown. The curves are similar to the curves found by authors in Reference [44].

In the first heating (black colour in Figure S3), from 25 to 180 °C, a very broad melting endotherm spanning the whole interval is found, with the main maximum at approximately 117 °C, and another smaller maximum at approximately 50 °C. The presence of the endotherm means that the material is phase separated with a pure crystalline phase.

In Reference [44] the first maximum is attributed to the melting of BA blocks, and the second maximum to the melting of BT blocks. We do not agree, and we consider that the whole endotherm is due to the melting of BT blocks, with two populations of crystal sizes that melt at different temperatures (lower for smaller crystals). The block length is very short, and for this reason the melting point for the BT blocks, 117 °C, is much lower than the melting point for high-molecular-weight poly(butylene–terephthalate) (PBT), approximately 225 °C [42,45]. For the short BA blocks, the same trend is expected, and taking into account that the melting point for high-molecular-weight poly(butylene adipate) (PBA) is in the range 50–60 °C depending on the crystal form (α or β) [46,47], the very short BA blocks are expected to melt at temperatures well below ambient temperature.

The melting enthalpy is found to be around 28 J·g^−1^. In order to calculate the crystallinity degree, the enthalpy value for 100% crystalline material has to be known. In the literature, a value of 114 J·g^−1^ for 100% crystalline PBAT is accepted [22,33,37,44,48]. However, if the origin of the value is tracked, it is found that most of the works refer to other works, with the result being that the value was theoretically calculated from ester, methylene and aromatic ring contributions [49]. This calculation assumes that all PBAT chain crystallizes, which is not correct. In this copolymer, the crystallization takes place in the phase-separated short blocks of BT, and the enthalpy value for 100% crystalline PBT experimentally calculated is 145 J·g^−1^ [50], a 27% higher value than the 114 J·g^−1^ assumed for 100% crystalline PBAT. The value for 100% crystalline PBT that is experimentally determined is close to the theoretically calculated value (150 J·g^−1^) [49]. Surprisingly, the authors in Reference [44], despite admitting that in the second heating step the melting endotherm is only due to butylene–terephthalate blocks, confirmed by X-ray patterns, use the calculated 100% crystalline PBAT value instead of the 100% crystalline PBT value.

We will use the value of PBT 100% crystalline enthalpy for the calculations, which will produce lower crystalline values than in the literature. From proton NMR, PBAT has a 48% mol of terephthalate, and therefore 50% of the weight of BT segments, although most parts of them are alternating with butylene–adipate segments instead of forming blocks, and will not be able to crystallize. The calculated crystallinity for the unirradiated PBAT, after processing and storage, is approximately 38% with respect to the total butylene–terephthalate in the copolymer, and 19% with respect to the total copolymer.

In the cooling step from the melt (red colour in Figure S3), a single broad crystallization peak is found with a minimum at approximately 70.5 °C, followed by a T_g_ at low temperature. The enthalpy of crystallization, with a value of approximately 22 J·g^−1^ is slightly lower than the melting enthalpy, because the material does not have enough time to develop a full crystallization.

In the second heating step (blue colour in Figure S3), a T_g_ is found at low temperature, approximately −34 °C, followed by an extremely wide melting endotherm (from 4 to 149 °C) with the maximum at approximately 120 °C. Usually, when the copolymer is precipitated from solution [44] or annealed from the melt, the melting point in the second heating step results in a lower melting temperature, but in this case, due to the processing in the melt with relatively fast cooling in the casting rollers, the formation of the crystals has been stopped and the melting point in the second heating step is higher. For the melting enthalpy, the value is similar to the crystallization enthalpy, as expected, and slightly lower than the melting enthalpy in the first heating step, because the material is not given enough time for the full development of crystallization. Still, the difference in crystallinity is not very high, a 19% reduction with respect to the first heating step, showing that in the testing conditions, most of the crystallinity is recovered, which differs from the authors from Reference [44], who found a much higher crystallinity reduction in the second heating step, when samples were cooled at −10 °C·min^−1^ from the melt, compared to our experiments. No cold crystallization was found.

Summarizing the thermal properties, only the butylene–terephthalate segments are able to crystallize, with melting at approximately 117 °C, well below (−108 °C approximately) that of the high-molecular-weight PBT. Due to the relatively short length of the butylene–terephthalate blocks, PBAT crystallization is low, 19% with respect to the total weight of the material, with a high recrystallization recovery from the melt, circa 80%. The amorphous phase, accounting for most of the material, produced a T_g_ at a low temperature, approximately −34 °C.

In Figure S4 in the Supplementary Materials, the tensile stress–strain curve for PBAT is shown.

The shape of the curve is typical for a tough thermoplastic, similar to polyethylene. After a fast increase in stress at low strain, producing a Young’s Modulus of approximately 38 ± 2 MPa, the material yields at approximately 44% strain and 9 MPa stress, with a slight decrease in stress afterwards, producing a neck, followed by a very narrow pseudo-plateau from 60 to 130% strain approximately, where stress does not change significantly with strain. Above 130% strain, stress increases constantly and linearly with strain until rupture at high strain, approximately 2100 ± 30%, reaching a high tensile strength of 41 ± 3 MPa. The calculated values for the mechanical properties show that, because the material is highly amorphous but with high molecular weight, it reaches high tensile strength and high strain at break with a relatively low modulus. Compared with the data found in the literature for PBAT with a similar terephthalate/adipate molar ratio (around 50:50) [19,44], the tensile strength and strain at break are higher and the Young’s Modulus is lower for the PBAT material in this work.

### 3.2. Gel Content

Gel content was measured to determine the degree of crosslinking caused by gamma radiation, producing a three-dimensional network that makes the polymer insoluble. PBAT samples were irradiated, by duplicate, at 0 (unirradiated) 10, 20, 30, 50, 100, 200, 500, 700, 1000, 1200, 1500 and 2000 kGy for gel content testing.

**Figure 2:**
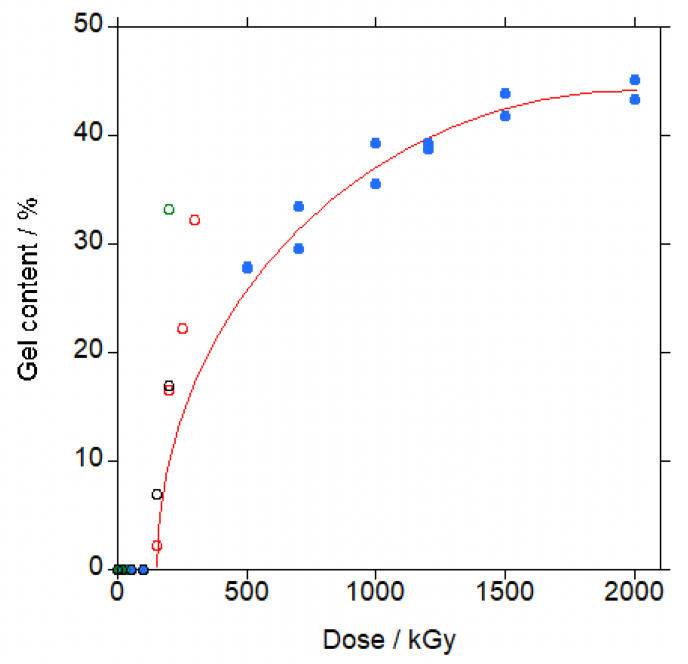
Gel content for irradiated PBAT vs. dose (filled circles).

In Figure 2, the data for the gel content of the irradiated samples of PBAT are shown (filled blue circles; the red line is not a curve fitting, it is just a guide to the eye).

Up to 100 kGy, no gel was found. For 200 kGy, gel was present, as proven by the impossibility to filter the solution because the filter clogged, and could not be separated. Above 200 kGy, the gel content initially increased rapidly, and more slowly at increased doses, with a maximum gel content of approximately 44% weight at 2000 kGy.

The gel content appeared in between 100 and 200 kGy, in agreement with the literature’s data (open circles in Figure 2) [19,26,27]. At increased doses, up to 200 kGy, the gel content seems to be higher for the PBAT copolymers in the literature, of an undisclosed terephthalate/adipate molar ratio except for one work with similar ratio (47.4% mol adipate) [19], for which the gel content was 33.2% at 200 kGy, significantly above our 500 kGy gel content of 28%. This difference could be due to a higher initial molecular weight of the PBAT in Reference [19] that would result in a higher gel content at the same dose, as found for PCL [39].

In Reference [27], the data for supposedly pure PBAT, are completely different from Figure 2, as it can be seen in Figure S5. Gel appeared already at 20 kGy and gel content was much higher at the same dose for the other data found in the bibliography. Furthermore, the same authors provided completely different data, for the same PBAT, in Reference [28] (see Figure S5). In addition, the trend is suspiciously similar to the trend found in other works where a crosslinker was added to PBAT, shown in Figure S6 [26]. Thus, clearly, the data from Reference [27] are obtained from a PBAT blended with a crosslinker although the authors do not mention any crosslinker in the paper and, therefore, gel data from Reference [27] cannot be compared with the data for pure PBAT.

**Figure 3:**
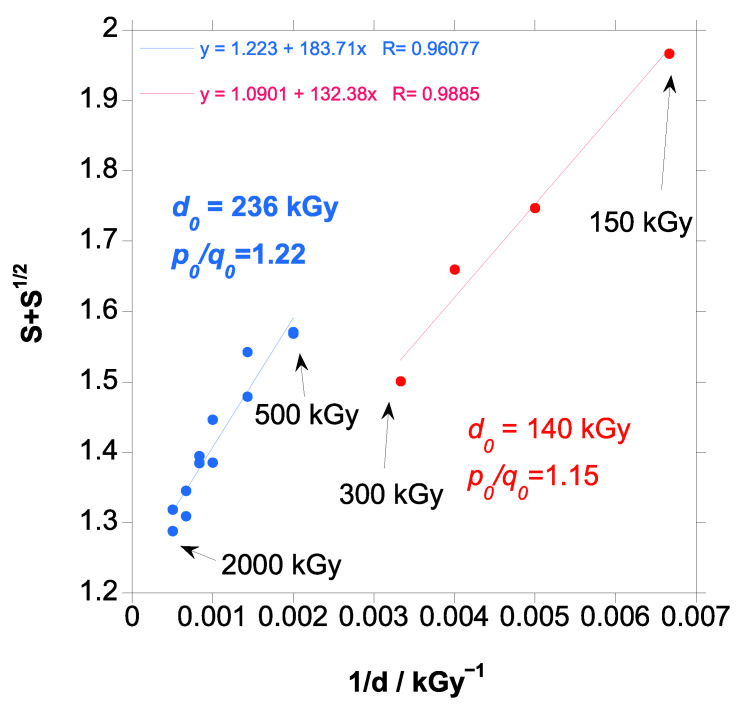
Charlesby–Pinner plot for PBAT irradiated in this work (blue circles) and PBAT from Reference [16] (red circles).

When the data were plotted in a classical Charlesby–Pinner graph, Figure 3 (blue line), a linear trend was observed.

From the Charlesby–Pinner equation, a minimum dose for gelation d_o_ of 236 kGy was calculated, which is slightly higher that the experimental value found (in the range 100 to 200 kGy) and the value of 140 kGy found in the literature [19] for a PBAT with similar adipate content (47.4% mol) as the PBAT in this work.

A linear fitting means that crosslinking is at random, and that the crosslinking ratio does not change when the material crosslinking increases with the increase in dose. Deviation from linearity in a Charlesby–Pinner plot indicates that crosslinking events are occurring in a less random and more directed manner, and, therefore, when crosslinking increases, the efficiency of further irradiation to crosslinking increases. In this case, it is usual to fit to a straight line the data at lower doses [30,51]. If the data are compared with the data from the literature [19], Figure 3 (red circles), it can be clearly seen that the missing data in our work from 150 to 500 kGy are key to determining precisely the minimum dose of gelation (intercept with s + s^1/2^ = 0). The higher gel content found for the sample in Reference [19], attributed to a higher initial molecular weight of the PBAT, allowed for a more precise calculation of d_o_ on the Charlesby–Pinner with the gel content data at lower doses than in our study. If our data from 500 kGy to 2000 kGy were slightly curved with respect to the missing data from 150 to 500 kGy, the minimum dose for gelation would be overestimated and would explain the difference with the experimental value found (in the range 100 to 200 kGy) and the value of 140 kGy given in the literature.

The value for the crosslinking efficiency (q_o_/p_o_) of 0.82 is similar to the value of 0.87 found in the literature [19]; thus, chain scission slightly predominates over crosslinking.

### 3.3. Molecular Weight

Size Exclusion Chromatography (SEC) was used to determine the changes in molecular weight caused by gamma irradiation. PBAT samples were irradiated at 0 (unirradiated) 10, 20, 30, 50, 100, 200, 500, 700, 1000, 1200, 1500 and 2000 kGy for molecular weight determination. From 0 to 100 kGy, the measured molecular weight corresponds to the whole sample, whereas at 200 kGy and above, because of the existence of a crosslinked and therefore insoluble material, the measured molecular weight corresponds only to the soluble part of the material.

**Figure 4:**
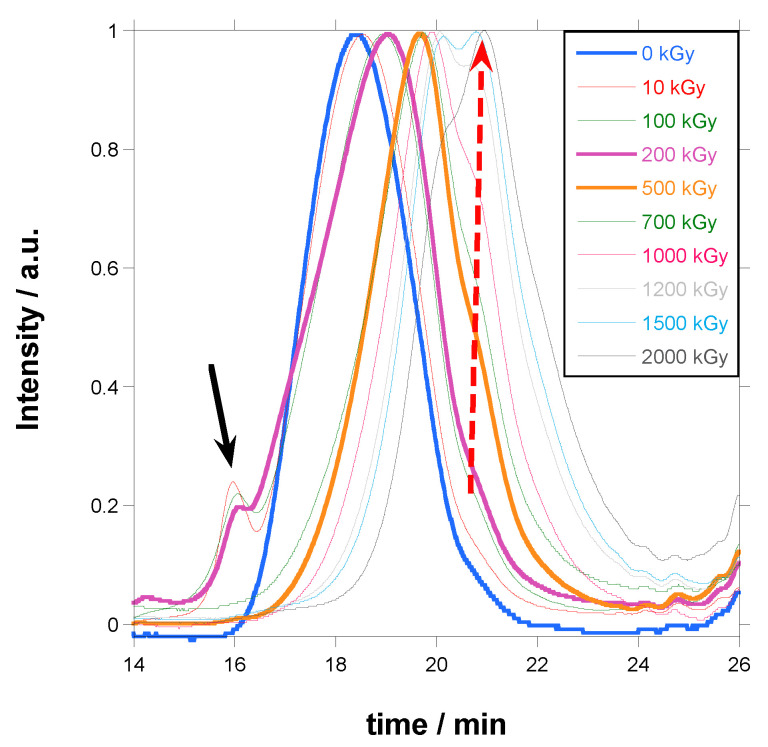
SEC curves for PBAT irradiated at different doses.

In Figure 4, the normalized curves for irradiated PBAT are shown.

Several details can be appreciated in the graph. The main peak (M_p_), initially at 82,000 Da for the unirradiated PBAT, moves to longer times and lower molecular weights when irradiation dose increases, which could lead one to think that molecular weight decreases continuously. However, a small peak at short times due to chains of molecular weight higher than the exclusion limit of the columns (pointed out by a black arrow in Figure 4) appears at 10 kGy and completely disappears at 500 kGy. At high irradiation doses, a shoulder at long times due to low molecular weight chains develops to a peak at 1200 kGy and becomes the main peak in 2000 kGy (pointed out by a red dashed arrow in Figure 4), with a molecular weight of 8700 Da. At the same time, the width of the peak increases with the increase in dose.

These results can be explained by the crosslinking of molecules, producing branching that increases molecular weight (peak at shorter times), and by the simultaneous scission of chains that produces shorter fragments and therefore a decrease in molecular weight (peak at longer times). Both effects lead to a broadening of the molecular weight distribution.

**Figure 5:**
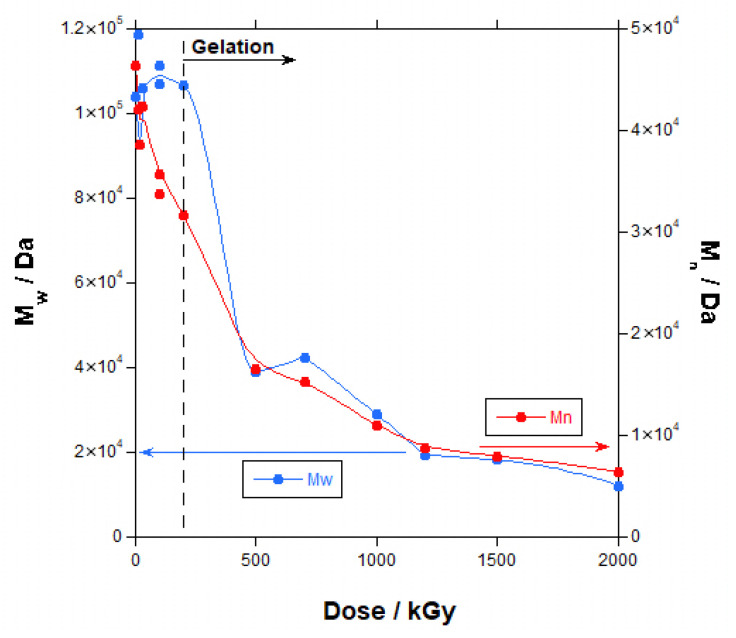
Molecular weight for irradiated PBAT (number average: red circles; weight average: blue circles) vs. dose.

**Figure 6:**
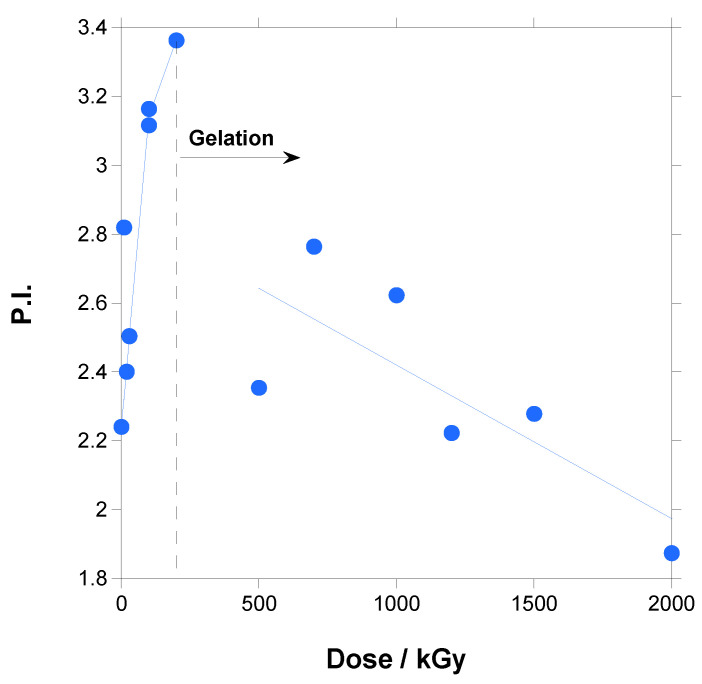
Polydispersity index for irradiated PBAT vs. dose.

These conclusions can be better justified when the data for the number average molecular weight (M_n_), weight average molecular weight (M_w_) and polydispersity index (PI) are represented, as in Figure 5 and Figure 6 (the lines are not curve fittings; they are just a guide to the eye).

Until gelation, M_n_ decreased continuously with the increase in dose, whereas M_w_ did not change significantly with the increase in dose and then decreased. The polydispersity index, being the ratio M_w_/M_n_, logically increased.

Thus, until gelation, branching produced by crosslinking reactions that increase molecular weight compensated the decrease in molecular weight caused by chain scission, leading to a fairly constant M_w_ with the increase in dose, whereas the shorter chains caused by chain scission dominated the M_n_ value with a decrease with the increase in dose. Additionally, there was a simultaneous increase in high-molecular-weight and low-molecular-weight species, produced an increase in polydispersity with the increase in dose. In other words, the occurrence of intermolecular crosslinking that generates high-molecular-weight branched or networked structures, coexists with degraded chains, broadening the distribution. After gelation, only the fragments produced by scission are soluble and could be measured by SEC, and a decrease in molecular weight was found as a consequence of the increase in the fraction of low-molecular-weight species.

Only two works in the literature measured molecular weight by SEC in irradiated PBAT. In one of them [29], M_n_, M_w_ and PI increased with the dose until a maximum of 90 kGy, and, in the other work [32], M_n_, M_w_ and PI stayed fairly constant until a maximum of 100 kGy. In both cases, measurements were taken before gelation. At these low doses, as seen in Figure 5, M_n_ and M_w_ present certain dispersions, and this could explain the difference in results. When compared with the results for other polymers, our results for PBAT are similar to the results found for other polyesters such as the aliphatic polycaprolactone [39] and poly(butylene succinate-co-adipate) [52], and for a poly(ether-urethane) [51].

### 3.4. Proton NMR

**Figure 7:**
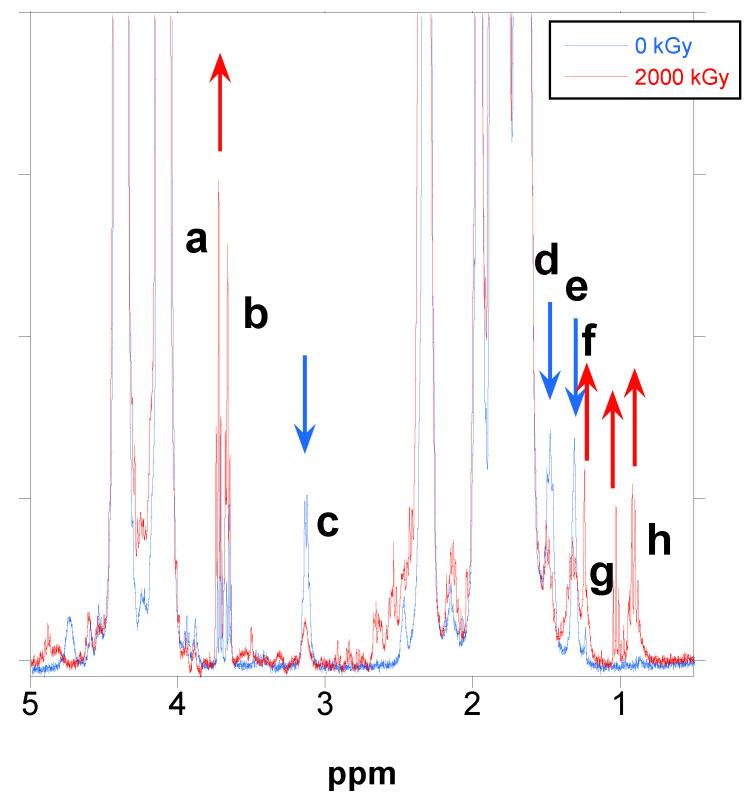
Proton NMR spectra for unirradiated PBAT and PBAT irradiated at 2000 kGy magnified in the aliphatics region.

In the following figure (Figure 7), the changes in the proton NMR spectra between the unirradiated PBAT and the soluble part of PBAT irradiated at 2000 kGy are shown. In Figure S2 in the Supplementary Materials, the main peaks for PBAT are described. However, some other minor peaks are found in the spectrum that are important for the comprehension of the interaction of gamma radiation with PBAT.

As seen in Figure 7, there are some minor peaks in the aliphatics region. These peaks (blue spectrum in Figure 7) are the following:

- Triplets at 3.73 (a) and 3.66 (b) ppm;
- Quadruplet at 3.14 (c) ppm;
- Multiplets at 1.48 (d) and 1.32 (e) ppm.

The triplets at 3.73 and 3.66 ppm are due to terminal methylene being linked to a hydroxyl group (-CH_2_-OH), for terephthalate (-T-Bu-OH) and adipate (-Ad-Bu-OH) respectively. This triplet can be seen for PBAT in Reference [44] (Figure S7 in the Supplementary Materials), although the authors did not mention it.

The rest of the signals (c, d, and e), are assigned to the protons of hexamethylene diurethane. Diisocyanates such as hexamethylene diisocyanate (HDI) have been used as chain extenders in hydroxyl-terminated polymers to increase their molecular weight [53,54]. The signals for hexamethylene diurethane when PBTA/PEG diols are chain extended [53] or when PCL is chain extended [54] are coincident with the signals in Figure 7 for unirradiated PBAT, as seen in Figure S8 in the Supplementary Materials. In addition, the signals are also coincident with the signals for hexamethylene diurethane in a polycarbonate diol chain extended with HDI synthesized at our laboratory, shown in Figure S9 in the Supplementary Materials.

The proton–proton homonuclear correlation spectrum confirmed that signals (c), (d) and (e) are correlated.

As seen in Figure 7 (red spectrum), the signals related to terminal hydroxyl groups increased with irradiation, whereas the signals for the hexamethylene diurethane groups decreased with irradiation. In addition, new signals appeared at 1.24 (f), 1.03 (g) (triplet) and 0.92 (h) (multiplet) ppm, related to terminal aliphatic chains such as butyl (-O-CH_2_-CH_2_-CH_2_-CH_3_).

When the integrals of the signals were measured and normalized to the number of protons in each signal, it was found that the ratio of the signals adipate/butylene (A/B) was almost constant with the increase in dose, whereas the ratio of aromatic signals for terephthalate to adipate (T/A) or butylene (T/B), increased slightly (5–6%); thus, the soluble part of PBAT after gelation was enriched for terephthalate, which would be consistent with the preferential rupture of bonds in the adipate units.

**Figure 8:**
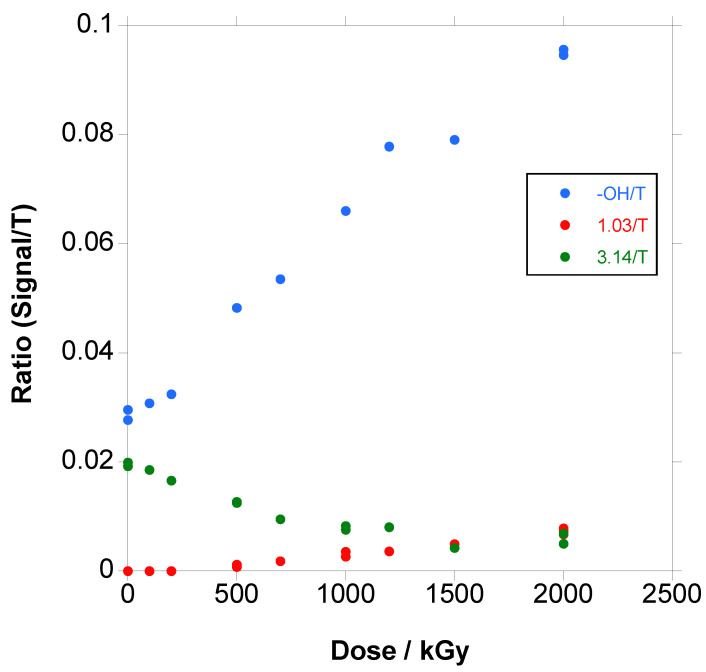
Ratio of the normalized signals (a + b) (blue colour), c (green colour) and g (red colour) to the terephthalate signal.

If the ratio of the methylene linked to hydroxyl (a) + (b), the signal at 3.24 ppm for hexamethylene diurethane (c) and the signal at 1.23 (g) for aliphatic terminal chains to the terephthalate signal, is plotted, as in Figure 8, the terminal hydroxyl groups are apparently the main groups formed with irradiation, with an increase of almost 250% at 2000 kGy, and, at the same time, the diurethane groups disappear and aliphatic terminal chains appear with the increase in dose, although in less proportion.

It is very important to point out that the ratio of the groups does not vary significantly with the increase in dose when gelation is reached and only the soluble part of the polymer is analyzed above gelation (see Figure 7); thus, it seems that the mechanism of degradation is independent of the dose.

**Figure 9:**
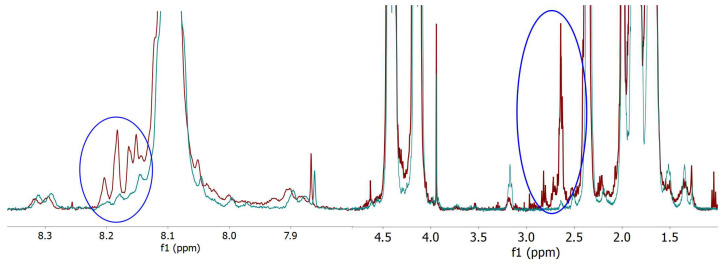
Proton NMR spectra for derivatized unirradiated PBAT (blue curve) and PBAT irradiated at 2000 kGy (red curve), magnified in the aromatics region (**left**) and the aliphatics region (**right**) with the same intensity scale.

When the unirradiated PBAT and the PBAT irradiated at 2000 kGy are in situ derivatized with trifluoroacetic anhydride, the hydroxyl groups are esterified and the carboxylic groups form anhydride groups. In Figure 9, the spectra for the derivatized materials are shown.

The signals for terminal hydroxyl groups, because they are esterified, were submerged in the big signals at 4–5.5 ppm and could not be seen, and new signals appeared in the aromatic region (Figure 9 left) and in the aliphatics region (Figure 9 right). These new signals at above 8.1 ppm and at 2.64 ppm are consistent with the formation of terephthalic and adipic anhydrides by reactions with trifluoroacetic anhydride. The aromatic signals are partially submerged in the big terephthalate signal and cannot be integrated separately, but the signal at 2.64 ppm can be integrated. The integral for the 2.64 ppm, taking the adipate signal at 2.3 ppm as a reference for normalization, is almost a 50% bigger that the signal for hydroxyl group. If we add the signal for aromatic carboxylic groups, it is clear that the rupture of the -COO-CH_2_ bond is more likely than the rupture of the -CO-O bond, and both seems to be the preferred broken bonds. The rupture of these bonds should produce a corresponding amount of aliphatic chain ends, but the signals f, g and h (Figure 7) are much smaller. This result is explained by the preference of the generated aliphatic radicals (-CH_2_·) to recombine with another radical from another chain leading to a branching point (crosslink) instead of abstracting a hydrogen radical to produce a saturated chain end. These crosslinks will finally produce the insoluble network and will not be detected in the soluble part of the material.

**Figure 10:**
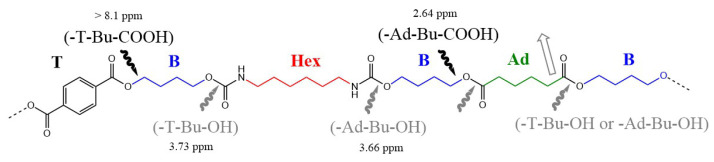
Preferential PBAT bonds broken by gamma irradiation.

From these data, it is proposed that the links between the ester group and the aliphatic chain, leading to carboxylic groups, and the ester and urethane bonds, leading to hydroxyl groups, are preferentially broken by gamma radiation, as shown in Figure 10.

### 3.5. Thermal Properties

Because gamma irradiation causes structural changes at the molecular level, it can have a substantial impact on the crystallinity of PBAT. PBAT samples were irradiated at 0 (unirradiated) 10, 20, 30, 50, 100, 200, 500, 700, 1000, 1200, 1500 and 2000 kGy for thermal properties evaluation. Differential Scanning Calorimetry (DSC) was used to examine how gamma radiation affected the thermal behaviour of PBAT. In the first heating step, from 25 °C to 180 °C at 10 °C min^−1^, the melting point and the crystallinity of the irradiated material were calculated. The crystallinity of the material is one of the most important parameters influencing mechanical properties, and therefore it is important to evaluate this crystallinity to relate it with the determined tensile properties. In the second and third step, cooling from 180 °C to −80 °C at a rate of −10 °C min^−1^ and heating from −80 °C to 180 °C at 10 °C min^−1^, respectively, the effect of irradiation on the crystallization, glass transition temperature and melting was evaluated.

**Figure 11:**
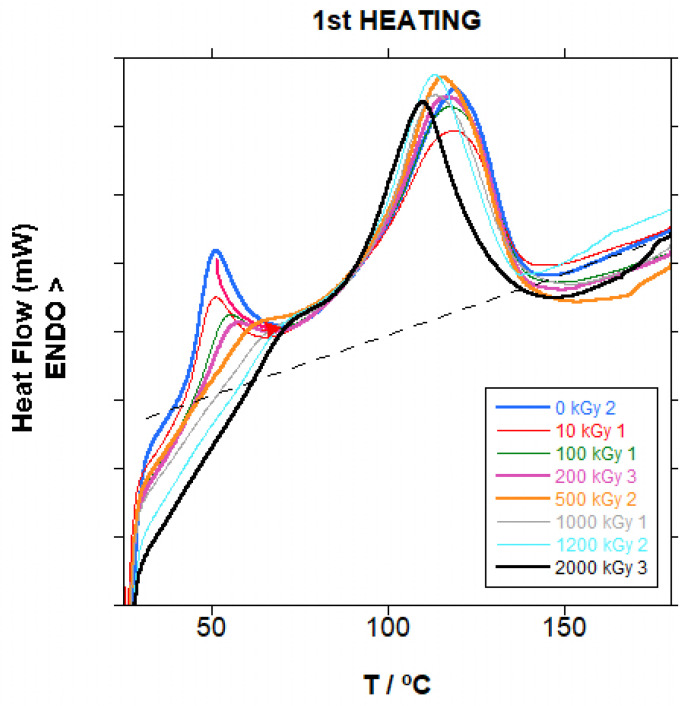
First heating curves for PBAT irradiated at different doses. Dashed line is an example of the baseline used for melting enthalpy calculation (0 kGy sample).

**Figure 12:**
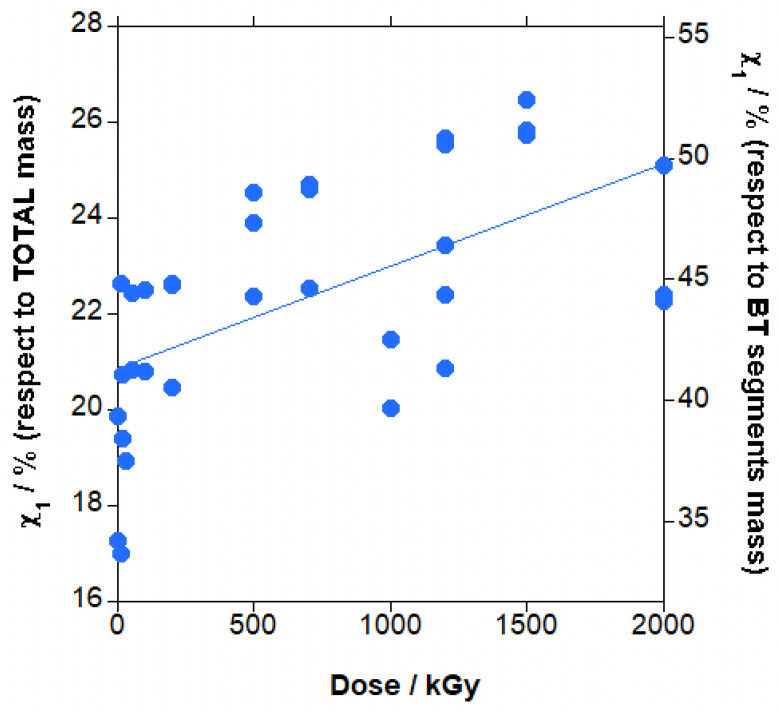
Crystallinity in the first heating step (χ_1_) for irradiated PBAT vs. dose.

In Figure 11, the curves for the first heating cycle for PBAT irradiated at different doses are presented. The first heating cycle provides the degree of crystallinity of PBAT, a very important parameter for the mechanical properties. Before irradiation, the curve presents a main peak (T_m1_) at approximately 118 °C, and a smaller peak (T_m0_) at lower temperatures, 51 °C approximately. When the irradiation dose increased, the main peak slightly shifted linearly to lower temperatures (−7 °C approximately) whereas the smaller peak shifted to slightly higher temperatures and finally disappeared in the main peak, as seen in Figure S10 in the Supplementary Materials. The dispersion of T_m1_ and T_m0_ results is relatively low and the trends are considered significant. The melting peak is very broad, from above ambient temperature to around 145 °C, which makes it difficult to draw the baseline (dashed line in Figure 11) and leads to the very wide dispersion of the data, as seen in Figure 12, for the calculated crystallinity (χ_1_). A trend to slightly increase the crystallinity is found, although because of the dispersion of the data, the trend cannot be considered significant.

It is accepted that ionizing radiation affects more strongly the amorphous phase than the crystalline phase in semicrystalline polymers, and that crystals are affected, especially in the boundaries of the crystals. If we consider that the higher the melting point, the bigger and more perfect are the polymeric crystals, the lower temperature peak is due to small crystals with a larger interface with the amorphous phase. Additionally, the disappearance of this peak shows that these smaller crystals, with a higher surface/volume ratio (that is, a larger boundary than bigger crystals), are more strongly affected than the larger crystals melting at higher temperatures, which are also affected, as shown by the decrease in the melting temperature. The predominant effect of radiation is chain scission, as shown by the Charlesby–Pinner analysis, and the resulting shorter chains, with higher mobility, can rearrange into larger crystals, resulting in the slight increase in crystallinity. If crosslinking was dominant, the crosslinked chains would not be able to align and pack into a regular crystalline lattice and crystallinity would decrease with the increase in dose. The amount of crystallized BT sequences in PBAT copolymer is relatively high, 40–50% weight, taking into account the copolymer structure of PBAT, which makes more difficult the arrangement of these sequences into crystals. For the total amount of material, crystallinity is low, at 20 to 25% weight.

After melting PBAT, its thermal history is erased, and the material can be studied in a standardized manner with the same conditions, at the cooling and the second heating steps.

**Figure 13:**
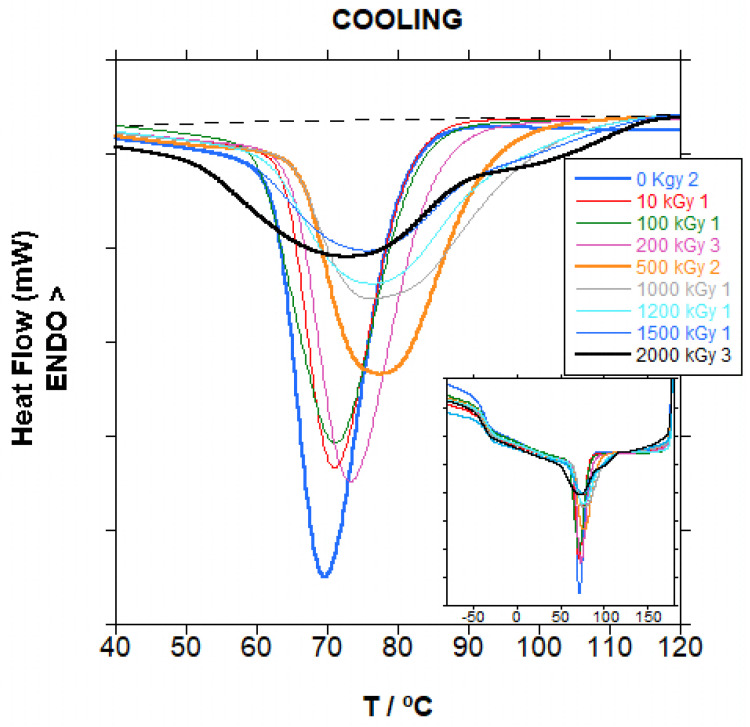
Cooling curves for irradiated PBAT at different doses magnified in the crystallization peak. Inset: the entire curve. Dashed line is an example of the baseline used for crystallization enthalpy calculation (2000 kGy sample).

When irradiated PBT is cooled from the melt, as in Figure 13, the effect of radiation in the amorphous region is reflected. Because the chain scission and crosslinking taking place in the amorphous region are distributed to the complete material in the homogeneous melted state, crystallization behaviour is modified. As seen in Figure 13, the temperature of the crystallization peak, initially at approximately 71 °C, increased and the peak became broader with the increase in dose. And at very high doses, above 500 kGy, a shoulder appeared at higher temperatures, and the peak became even much broader and developed into a second peak at 2000 kGy.

**Figure 14:**
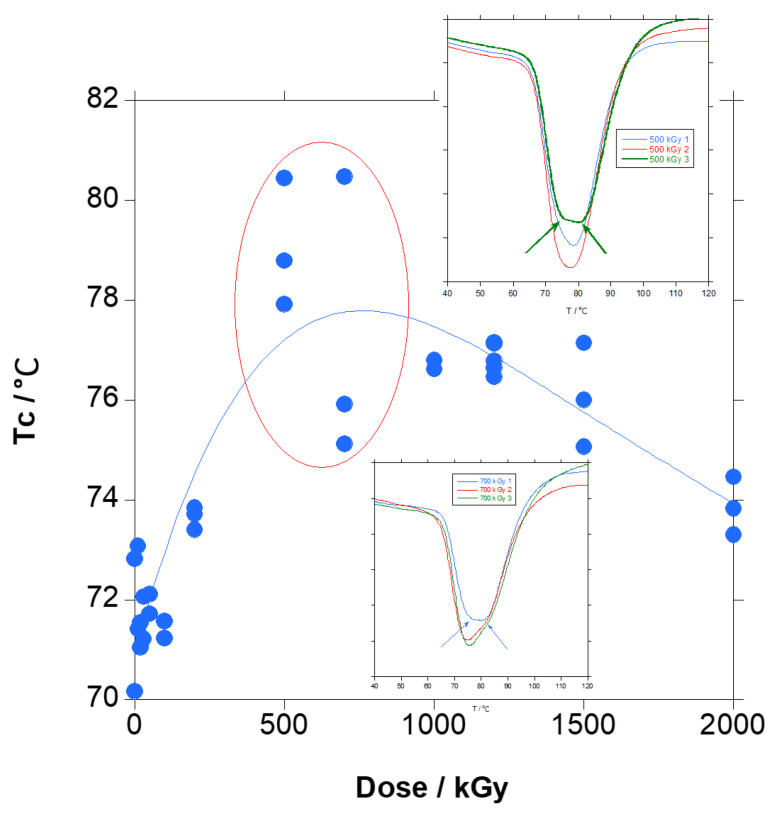
Temperature on the minimum of the crystallization peak (T_c_) for irradiated PBAT at different doses. Insets: top—crystallization peak for 500 kGy samples; bottom—crystallization peak for 700 kGy samples. The blue line is not a curve fitting, it is just a guide to the eye.

When the temperature of the crystallization peak is plotted vs. the dose, as in Figure 14, despite the dispersion due to the difficulty to draw the base line, it seems that the peak temperature increased slightly until approximately 500 kGy and then it slightly decreased, a trend that we consider significant. Up to 500 kGy, when the amount of gel is already significant, the effect of chain scission dominates, and crystallization is favoured by the shorter chains. At 500 and 700 kGy, the dispersion is at its maximum. If the peak is looked at closer, shown in the insets in Figure 14, it seems that the maximum is composed of two similar peaks with close maxima, and sometimes the maximum of the peak at lower temperature slightly dominates and sometimes the maximum of the peak at higher temperature slightly dominates. The presence of two populations of crystals is coincident with the appearance of gelation. When gelation occurs, part of the material, which is crosslinked, crystallizes with more difficulty, resulting in the peak at lower temperatures, whereas part of the material, not crosslinked, and with shorter chains due to chain scission, crystallizes more easily, resulting in the peak at higher temperatures. When the dose increases at even higher values, the amount of crosslinked material increases and the degree of crosslinking increases, and the amount of not-crosslinked material decreases, and the chains became even shorter. Thus, the crystallinity is further delayed and favoured respectively, and the crystallization peaks are more separated with the integral biassed to the crosslinked material, and therefore the maximum shifts to lower temperatures.

The opposite effect of crosslinking and chain scission did not produce a significant effect in the amount of crystallinity. In Figure S11 in the Supplementary Materials, it can be seen that for crystallinity in the first heating step dispersion is very high, which makes the trend statistically not significant, and the value for crystallinity is virtually unchanged with dose; thus, the effect of both effects compensates in the material crystallized from the melt.

The value for crystallinity in the cooling step is lower than crystallinity in the first heating step, as expected, because the material is not given enough time to fully develop its crystallinity before T_g_ is reached, freezing the material.

**Figure 15:**
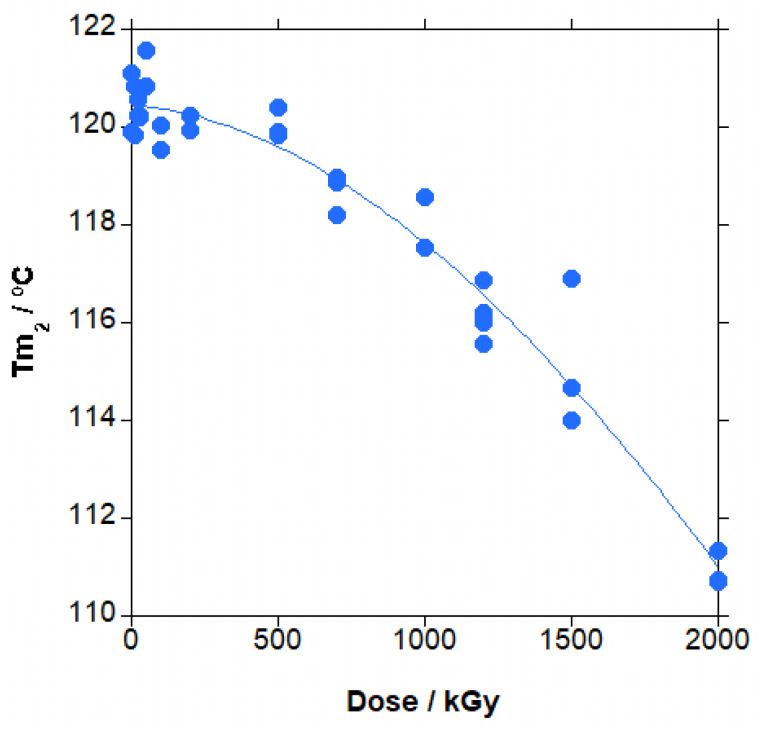
Main melting peak (T_m2_) in the second heating step for irradiated PBAT vs. dose.

In Figure S12 in the Supplementary Materials, the curves for the second heating for PBAT vs. irradiation dose are shown. After the T_g_ at low temperature, around −33 °C, an extremely broad melting endotherm, spanning from 0 to 150 °C, is found, with a maximum at approximately 112–120 °C and a secondary maximum at around 70 °C. The main melting peak decreased with temperature, whereas T_g_ apparently did not change significantly. The secondary melting maximum, because main melting peak shifted at a lower temperature, eventually became a shoulder. The extremely broad melting endotherm indicates that the formed crystals are extremely broad in size. In Figure 15 and Figures S13 and S14, the changes in T_g_, the maximum of the melting peak, and the percentage of crystallinity vs. irradiation dose are shown respectively.

T_g_ values show a dispersity on the data of 2–3 °C, and remain virtually unchanged with the increase in dose. In gel data, crosslinking increased with dose, which would increase the T_g_. Crystallinity, as shown in Figure S14, is almost constant and only at very high doses decreases slightly; thus, only at very high doses would it decrease the T_g_ very slightly. Additionally, chain scission would generate shorter chains with increased mobility that would decrease T_g_. These facts lead to the conclusion that the increase due to crosslinking and the decrease due to chain scission compensates and T_g_ remains unchanged.

The main effect on the second heating cycle is found for the maximum of the melting peak, as in Figure 15, with a very significant decrease (9 °C at 2000 kGy; a bit larger than for the first heating cycle) above 500 kG with the increase in dose (the blue line is not a curve fitting, it is just a guide to the eye.). This effect is due to both crosslinking and chain scission. Crosslinking makes more difficult the arrangement of the chains to form crystals, and chain scission leaves shorter chains and a larger amount of chain ends that do not enter crystals, producing smaller crystals that melt at lower temperatures.

Crystallinity, as in Figure S14, with a high dispersion of the data, only shows a slight, not significant decrease at very high doses. The amount of crystallinity in the second heating cycle is very low, around 17% of the weight of the material, similarly to the crystallinity in the cooling step. The reason is the same as is given for the cooling step: because the material is not given enough time to fully develop its crystallinity before T_g_ is reached, freezing the material.

In summary, for the PBAT irradiated in the solid state, the boundaries of the crystals are affected by chain scission leaving smaller crystals and shorter chains with higher mobility that crystallize more easily, leading to a significant melting point decrease and a slight increase in the amount of crystallinity (first heating step). From the melt (cooling step), the crystallization temperature is mainly influenced by chain scission until gelation and increases, and then it is mainly influenced by crosslinking and decreases. The amount of crystallization is virtually unchanged, with both effects compensating for each other. After crystallization, T_g_ is virtually unchanged (Figure S13; high dispersion of the data; trend statistically not significant), chain scission and crosslinking decrease the size of the crystals and therefore the melting point, and crystallinity is almost unchanged because the effect of crosslinking and chain scission compensate, as for T_g_.

When our results are compared with data in the literature for the melting point in the first heating cycle, the results in the literature (Figure S15 in the Supplementary Materials) showed a similar dispersion to our data with no trend at the ranges measured, up to 100 [32] and 300 kGy [19], and the same for the melting enthalpy (Figure S16 in the Supplementary Materials) [19]. The absolute values for melting point are higher and for melting endotherm are much lower for the PBAT with slightly lower adipate content (47.2% mol vs. our 52% mol) in Reference [19], which could be due to differences in the thermal history, in the block length for BT sequences and in molecular weight.

In the only work measuring crystallization [24], with a maximum dose of 50 kGy, crystallization temperature decreased (5.5 °C) and crystallization enthalpy increased (3.5 J·g^−1^) (Figure S17 in the Supplementary Materials). In the same range, it was not possible to find a trend in our results, with a maximum dispersion of the data of 3 °C for the crystallization temperature and 2 kJ·g^−1^ for the crystallization enthalpy, of the order of the maximum change in the literature data. We have shown that dispersion of the data is high, and authors in Reference [24] only performed a measurement per dose; thus, we think that the trends in Reference [24] cannot be taken as real, and more samples per dose and higher doses have to be measured, as in our work, to be able to recognize any trend on the data.

In the second heating cycle, the T_g_ value (Figure S18 in the Supplementary Materials), measured by DMTA and not DSC did not significantly change in doses up to 300 kGy [19,28], in agreement with our results, with the absolute values higher for the literature data due to the difference in the experimental technique. For the melting point (Figure S19 in the Supplementary Materials), starting at 120–123 °C at 0 kGy, similarly to our value of approximately 121 °C, its value decreased by 1.5 °C in doses up to 50 kGy [24] so that it is on the order of the maximum dispersion of our data (1.1 °C). We found that this decrease is significant only above gelation; thus, we think that with only one measurement for each dose, the decrease found in Reference [24] is not really significant. In doses up to 300 kGy [19], the melting point is similar for 0 and 100 kGy and for 200 and 300 kGy, with a sudden and very big decrease of 13 °C from 100 to 200 kGy. This result is absolutely abnormal. In our material, irradiated up to 2000 kGy (more than six times higher), the maximum decrease is 11 °C, and the decrease is progressive with dose. We think that, because of the difficulty on the drawing of the baseline, the calculated integrals in this reference are erroneous for 200 and 300 kGy. Finally, for the melting enthalpy, the literature provides absolute values much lower than in our results (8 to 13 J·g^−1^ vs. 25 J·g^−1^ respectively) and the trend is to increase in one case (3.5 J·g^−1^, 0 to 50 kGy) [24] and to decrease in other case (1.5 J·g^−1^ maximum, 0 to 300 kGy) [19] (Figure S20 in the Supplementary Materials). Our data, with a maximum dispersion of the data of 4.5 J·g^−1^, of the order of the changes in the literature, virtually do not change with dose up to very high doses; thus, again, we consider that with only one measurement at each dose and the limited range of irradiation, the contradictory trends found in the literature are not significant.

If the results for PBAT are compared with the results for other aromatic and aliphatic polyesters obtained by our group, such as aromatic PET (no gel up to 3000 kGy) [55], aliphatic PBS homopolymer (less than 20% gel at 400 kGy) and PBSA copolymer (30% gel at 400 kGy) [52], and aliphatic PCL (50% gel at 300 kGy for 80K molecular weight) [39], it is found that for the first heating cycle, melting temperature slightly decreased (PBS and PBSA) or remained constant (PCL) for the aliphatic polyesters and significantly decreased for the aromatic PET, as for aromatic PBAT, despite the difference in the crosslinking (not significant for PET with no gel up to 3000 kGy). The melting enthalpy for the first cycle remained constant (PBS; PBSA) or slightly increased (PCL; PET), as for PBAT (slight increase). In the second heating cycle, T_g_ remained constant (PBSA; PET) or slightly increased (PBS; PCL), melting temperature decreased for all, and melting enthalpy remained constant (PBS; PBSA) as for PBAT or slightly increased (PCL; PET). In the cooling cycle, crystallization enthalpy remained constant (PBS) as for PBAT or slightly increased (PBSA, PCL, and PET). It is noticeable that the trends in the thermal properties are very similar for polyesters irrespective of the chemical composition and the crosslinking/chain scission ratio.

The main difference is found in the crystallization temperature (T_c_), which decreased for the aliphatic polyesters and increased for PET, whereas for PBAT it increased until gelation and decreased after gelation. The difference in behaviour seems to be related to both chemical structure and gel formation. For PET, no gel is found and T_c_ increased because chain scission was highly predominant, and for aliphatic polyesters, T_c_ decreased before and after gelation. For PBAT, with aromatic rings in its chemical structure, and crosslinking efficiency of the order of the other aliphatic polyesters (0.82 for PBAT, 0.97 for PCL 80K, 0.64 for PBSA and <0.64 for PBS), the chain scission effect is predominant before gelation and the crosslinking effect is predominant after gelation.

It has to be pointed out that the shape of the cooling curve for PBAT is very similar to the copolymer PBSA, where aromatic terephthalate is substituted by aliphatic succinate and the adipate content is 26% mol (29% weight) instead of 52% mol (50% weight) in PBAT. When the irradiation dose increased, the crystallization peak split into two due to the fact that crosslinks are mainly in the amorphous PBA segments, whereas in homopolymers, crosslinking is homogeneous in the whole material.

### 3.6. Mechanical Properties

After PBAT was exposed to gamma irradiation at doses 0, 200, 500, 700, 1200, 1500, and 2000 kGy, its mechanical characteristics were examined. Young’s modulus, tensile strength (stress), and elongation at break (strain) were the three main mechanical characteristics that were measured.

**Figure 16:**
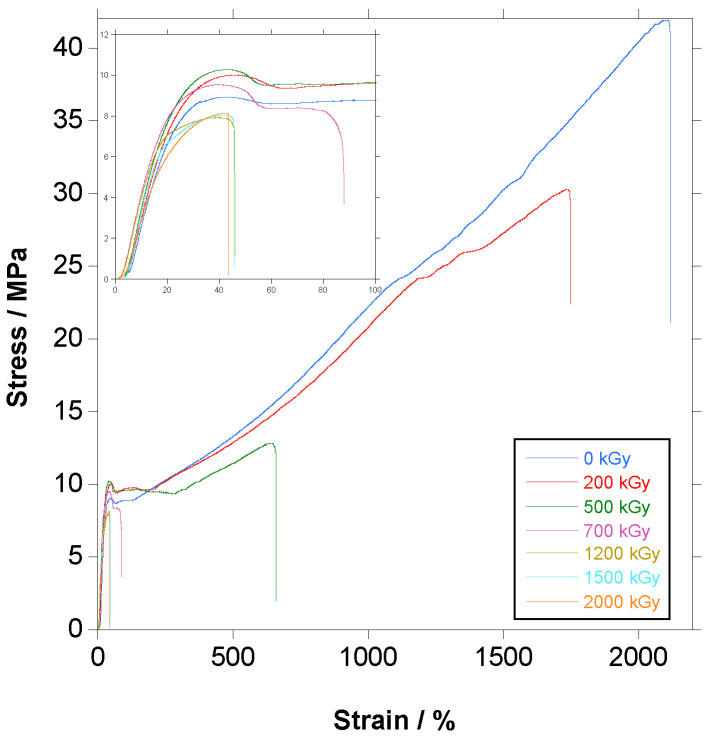
Stress–strain curves for PBAT irradiated at different doses (inset: magnification of the initial part of the curve).

In Figure 16, the changes in the stress–strain curves with the increase in dose are shown.

The shape of the curve did not change with respect to the unirradiated material, and the main effect was the decrease in strain leading to a decrease in the ultimate strength.

**Figure 17:**
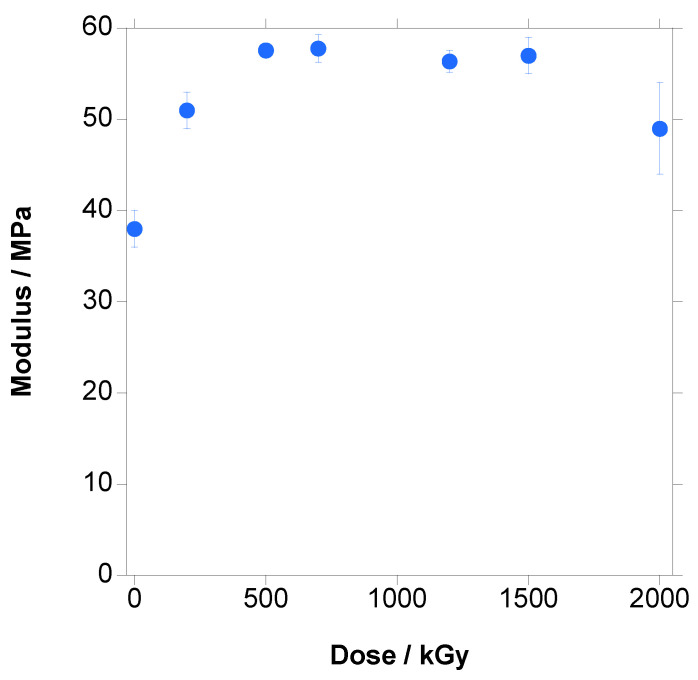
Young’s Modulus vs. dose for PBAT irradiated at different doses.

**Figure 18:**
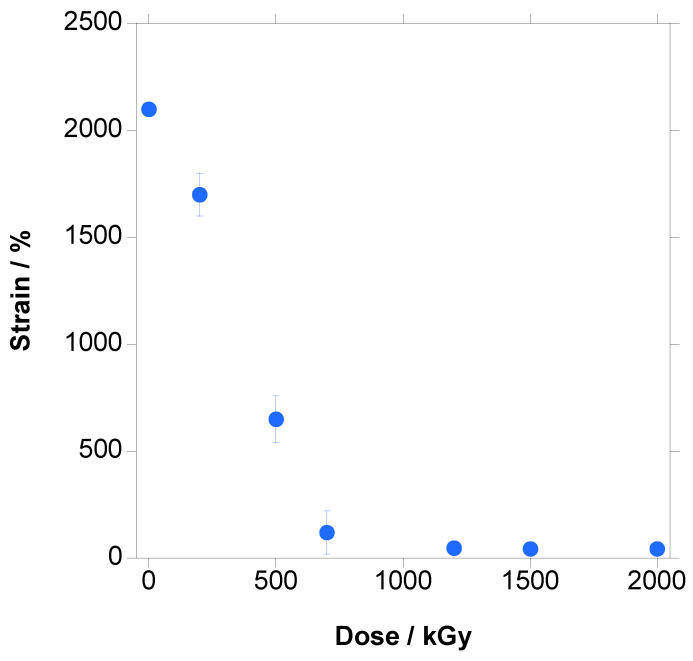
Elongation at break vs. dose for PBAT irradiated at different doses.

**Figure 19:**
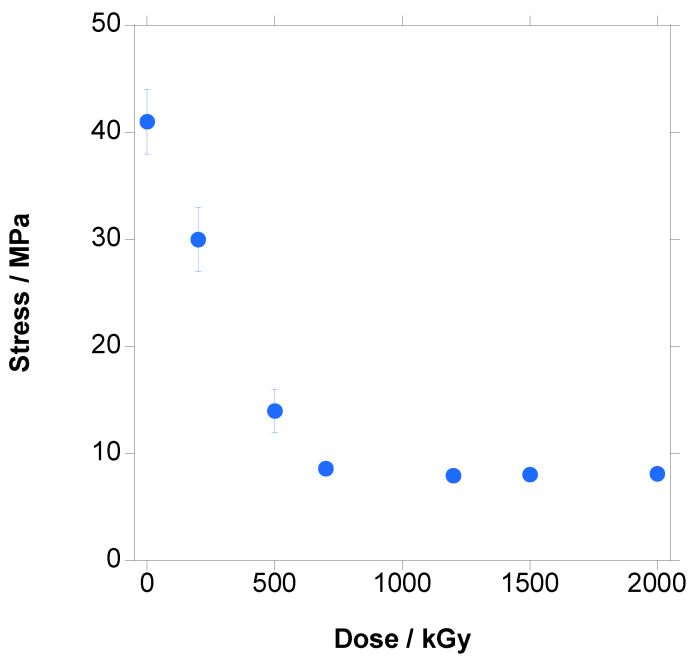
Tensile strength vs. dose for PBAT irradiated at different doses.

In Figure 17, Figure 18 and Figure 19, the variation in Young’s Modulus, ultimate strain and tensile strength with the increase in dose are presented, respectively. It has to be emphasized that crystallinity, which is a key parameter for mechanical properties, has to be taken from the first heating step because it is the crystallinity of the tested material.

Young’s Modulus, shown in Figure 17, increased initially and then remained constant, with an apparently slight decrease at 2000 kGy. This increase was related to the increase in crystallinity measured in DSC (Figure 12) that increases the stiffness of the material. If we look closely at Figure 12, the increase could be considered to be mainly up to 500 kGy, the dose for which a significant amount of crosslinked material is found, and at higher doses, crystallinity is practically constant, in coincidence with the trend of Young’s Modulus (Figure 17).

Elongation at break, shown in Figure 18, is a measure of a material’s ability to undergo plastic deformation before failure. It suffers a sharp, continuous quasi-linear decline from approximately 2100% for the unirradiated PBAT to less than 50% at 700 kGy. At doses above 700 kGy, the material becomes fragile and yielding is not reached any more. The effect on ultimate strain can be explained by the combined effect of crosslinking and chains scission, both deleterious for the strain. Crosslinking reduces the slippage of the chains when stretched, reducing the strain, and chain scission, which takes place preferentially in the amorphous phase and the boundaries of the crystals, reduces the connections between the crystals and amorphous phase, reducing the load-bearing capacity of the material and anticipating the rupture to lower strains.

The same trend is found for the tensile strength, shown in Figure 19, with a quasi-linear decrease until 700 kGy (from 41 to 8.6 MPa), followed by a constant value; the explanation is the same as for strain, pertaining to the combined harmful effect of crosslinking and chain scission.

When the literature’s data for PBAT are compared, Young’s Modulus, measured up to 300 kGy [19,24,29,31,32], does not show an increasing trend in any of the works (Figure S21 in the Supplementary Materials), as found for our results. Mostly, the trend was a fairly constant value or a slight decrease with the increase in dose. We have no explanation for the discrepancy, and we are fairly sure that our data and the trend are correct.

For tensile strength and ultimate strain, leaving apart the difference in absolute values that can be due to many factors (the adipate content in PBAT and testing procedure among them), when maximum dose is below 100 kGy (we have no data at these low doses), no obvious trend is found [24,29,31,32], but when the maximum dose is 200 [26] or 300 kGy [19], both parameters decreased with the increase in dose (Figures S22 and S23 in the Supplementary Materials), in coincidence with this study.

**Figure 20:**
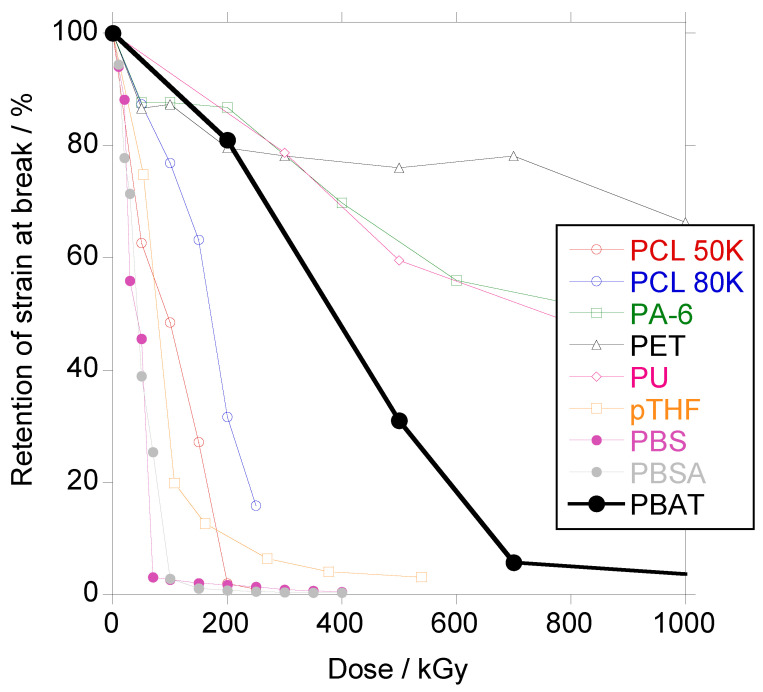
Retention of strain at break vs. dose for PBAT (black thick circles), PBS (purple circles), PBSA (grey circles), PCL 50K (red circles), PCL 80K (blue circles), PA-6 (green squares), PET (black triangles), aliphatic polyurethane (magenta diamonds) and poly(tetramethylene oxide) (orange squares).

In Figure S24 in the Supplementary Materials and in Figure 20, the results for tensile strength and elongation at break for PBAT, in terms of the retention of the property in comparison with other irradiated materials studied in the Elastomers’ group in Spain, are presented, respectively. PBAT shows a fairly good resistance to ionizing radiation, only below PU [55], PA-6 [56] and PET [57], and better than aliphatic polyesters (PCL, PBS, and PBSA) [32,52] and aliphatic polyether (pTHF) [51].

## 4. Conclusions

The irradiation of a commercial poly(butylene adipate-co-terephthalate) (PBAT) with 48% mol content of terephthalic units (50% weight of butylene–terephthalate sequences), 19% weight of crystallinity and good mechanical properties with gamma rays up to 2000 kGy produced a crosslinked material in the range 100–200 kGy and above. Charlesby–Pinner analysis proved that chain scission was slightly predominant over crosslinking.

Before gelation, irradiation generated branching that increased the molecular weight and chain scission decreased the molecular weight, producing an increase in dispersity with the increase in dose, as demonstrated by SEC.

The analysis by proton NMR proved that PBAT was chain extended with hexamethylene diisocyanate. Gamma irradiation produced the preferential rupture of -COO—CH2 bonds leading to the formation of carboxylic groups and, to a lesser extent, the rupture of -CO—OCH2 bonds leading to the formation of hydroxyl groups, whereas the radicals of the saturated aliphatic species led preferentially to crosslinking. Urethane bonds from chain extension are also broken by irradiation. The continuous variation in the chemical species before and after gelation showed that mechanism of degradation was independent of the dose and that the same species were produced in the whole material.

Thermal properties were differently affected depending on the predominant effect. Crosslinking took place mainly in the amorphous phase, whereas chain scission took place in the amorphous phase and the boundaries of the crystals, leaving smaller crystals and shorter chains with higher mobility that crystallize more easily, leading to a significant melting point decrease and a slight increase in the amount of crystallinity. Once the irradiated material was melted, the effect of chain scission predominated in the crystallization temperature until gelation and increased; for melting temperature, both crosslinking and chain scission produced a decrease, and for T_g_ and crystallinity, chain scission and crosslinking compensated, and remained virtually unchanged. These tendencies in PBAT were similar to tendencies in aromatic polyester PET except for the crystallization temperature, with the behaviour of PBAT being in between PET and aliphatic polyesters such as PCL, PBS and PBSA.

Irradiation influenced steadily the mechanical properties. Young’s Modulus followed the crystallinity changes, increasing until significant crosslinking was developed and then remaining constant. Stress at break and strain at break were continuously reduced with the increase in dose until a fragile material was obtained due to the combined effect of the crosslinking in the amorphous phase and the chain scission on the boundaries of the crystals.

The results in this work provide an understanding of the mechanism of crosslinking and chain scission in PBAT, and its relationship with the changes in molecular weight and thermal properties and with the continuous degradation of the mechanical properties until embrittlement, resolving the inconsistency of the results found in the literature and providing a framework for predicting the behaviour of this polymer when subjected to high-energy radiation.

## Data Availability

The raw data supporting the conclusions of this article will be made available by the authors on request.

## Supplementary Materials

The following supporting information can be downloaded at: https://www.mdpi.com/article/10.3390/polym18060683/s1. Figure S1: Part of the extruded and cast thin sheet from which test specimens were cut; Figure S2: Proton NMR spectrum of PBAT; Figure S3: Thermal behaviour of PBAT; Figure S4: Stress–strain curve for PBAT; Figure S5: Gel data for irradiated PBAT taken from Reference [27] (Orange circles) compared with data taken from Reference [26] (red circles), [28] (green circles; same authors as Reference [27]) and [19] (blue circles); Figure S6: Gel data for irradiated PBAT with 2% weight of crosslinker TAIC (black line with stars) (taken from Reference [26]); Figure S7: PBAT spectra from reference [44] with signals for terminal groups highlighted; Figure S8: Proton NMR signals for the hexamethylene diurethane (inside red circles) from References [54] (left) and [55] (right): Protons are not correctly assigned in Ref. [55]; Figure S9: Proton NMR signals for unirradiated PBAT (red colour) and for a polyurethane prepared from polycarbonate diol and hexamethylene diisocyanate (blue colour); Figure S10: Main melting peak (Tm1) and secondary melting peak (Tm0) in the first heating step for irradiated PBAT vs. dose; Figure S11: Crystallinity in the cooling step (χc) for irradiated PBAT vs. dose; Figure S12: Second heating curves for irradiated PBAT at different doses; Figure S13: Tg vs. dose for irradiated PBAT at different doses (second heating step); Figure S14: Crystallinity in the second heating step (χ2) for irradiated PBAT vs. dose; Figure S15: Melting temperature in the first heating cycle (Tm1) for irradiated PBAT (data taken from references [19,32]); Figure S16: Enthalpy of melting in the first heating cycle for irradiated PBAT (data taken from Ref. [19]); Figure S17: Temperature of crystallization (blue) and heat of crystallization (red) for irradiated PBAT (data taken from Ref. [24]); Figure S18: Glass transition temperature for irradiated PBAT by using DMTA analysis (data taken from References [19,28]); Figure S19: Temperature of melting in the second heating cycle for irradiated PBAT (data taken from References [19,24]); Figure S20: Melting enthalpy in the second heating cycle for irradiated PBAT (data taken from References [19,24]); Figure S21: Young’s Modulus for irradiated PBAT (data taken from References [19,24,29,31,32]); Figure S22: Tensile strength for irradiated PBAT (data taken from References [19,24,26,29,31,32]); Figure S23: Strain at break for irradiated PBAT (data taken from References [19,24,26,29,31,32]); and Figure S24: Retention of tensile strength vs. dose for PBAT (black thick circles), PBS (purple circles), PBSA (grey circles), PCL 50K (red circles), PCL 80K (blue circles), PA-6 (green squares), PET (black triangles), aliphatic polyurethane (magenta diamonds) and poly(tetramethylene oxide) (orange squares).
