# Supplementary material for: Effect of Gamma Radiation on the Chemical Structure and Physical Properties of Poly(butylene adipate-co-terephthalate)
Source: Polymers (Basel). 2026 Mar 11;18(6):683. doi: 10.3390/polym18060683 (PMC13030104; doi:10.3390/polym18060683)
Supplement: Supplementary file 1 [file polymers-18-00683-s001.zip › polymers-4184359-supplementary.pdf]

Supporting Information

Effect of gamma radiation on the chemical structure and physical properties of PBAT

Authors: Daniel Marcos Rios[1,§], Mohammed Amine Atrous[2,§], Guillermina Burillo[3], Abderrahmane Belhaoues[2], Rodrigo Navarro[1], Ángel Marcos-Fernández[1,*]

1. Institute of Polymer Science and Technology (ICTP-CSIC), Juan de la Cierva, 3, 28006, Madrid (Spain)
2. Process Engineering Department, Faculty of Technology, 20 August 1955-Skikda University, Skikda (Algeria)
3. Instituto de Ciencias Nucleares, Universidad Nacional Autónoma de México, Circuito Exterior s/n, Ciudad Universitaria, Delegación Coyoacán, C.P. 04510, CDMX (Mexico)

§ Mohammed Amine Atrous and Daniel Marcos Rios share first authorship.

* Corresponding author: amarcos@ictp.csic.es

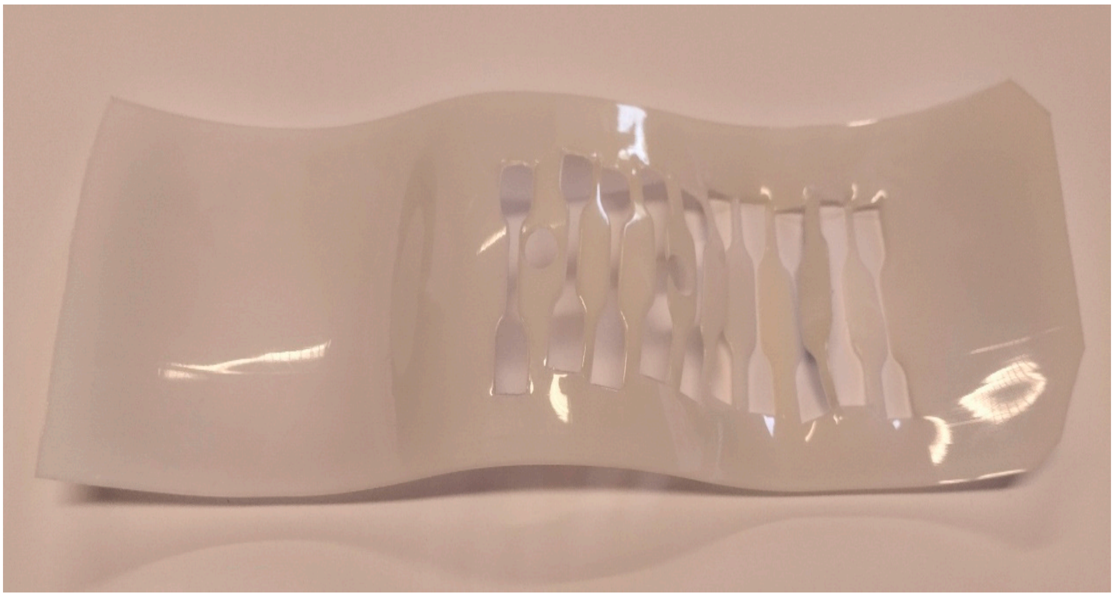

Figure S1: Part of the extruded and cast thin sheet from which test specimens were cut

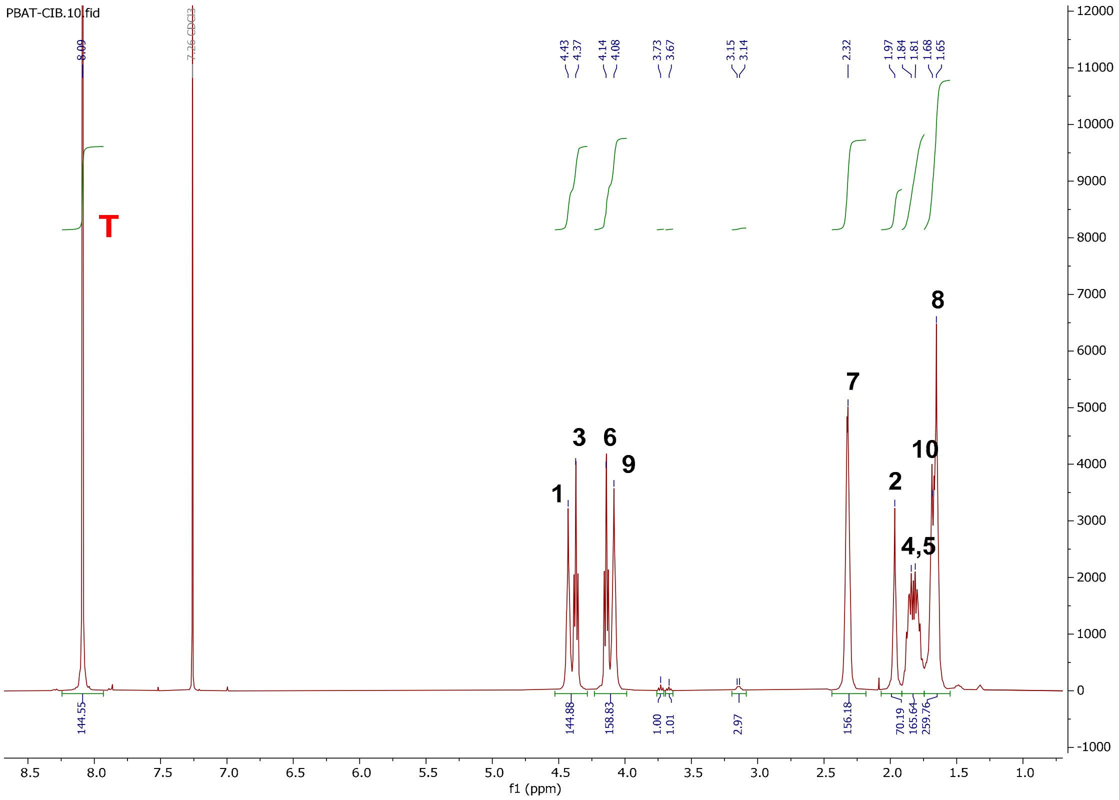

Figure S2. Proton NMR spectrum of PBAT

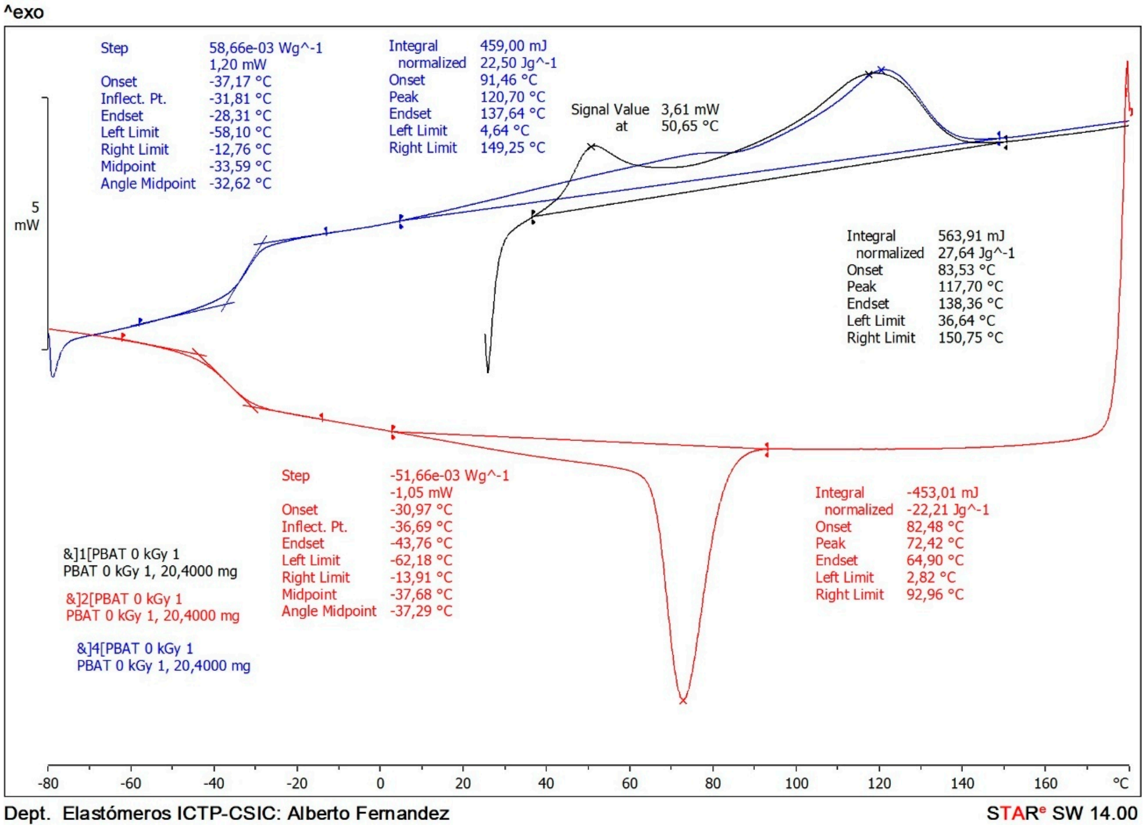

Figure S3. Thermal behaviour of PBAT

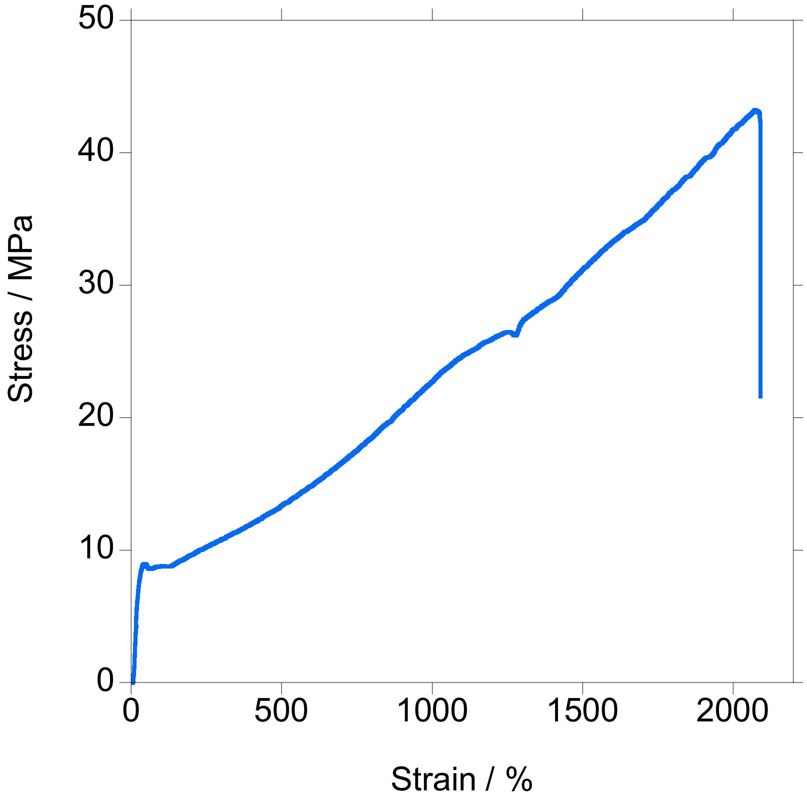

Figure S4. Stress–strain curve for PBAT

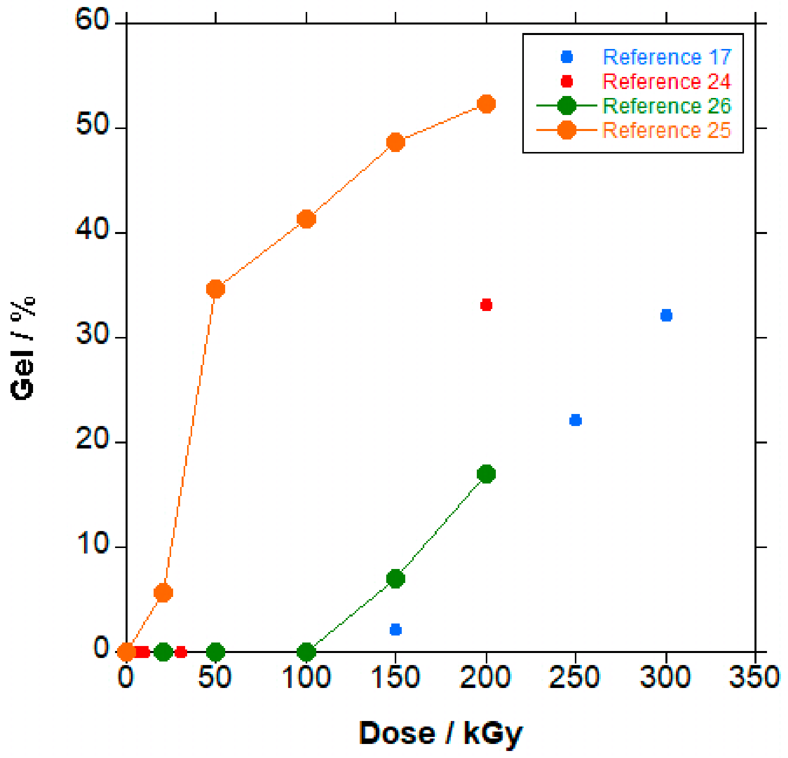

Figure S5. Gel data for irradiated PBAT taken from Reference [27] (Orange circles) compared with data taken from Reference [26] (red circles), [28] (green circles; same authors as Reference [27]) and [19] (blue circles)

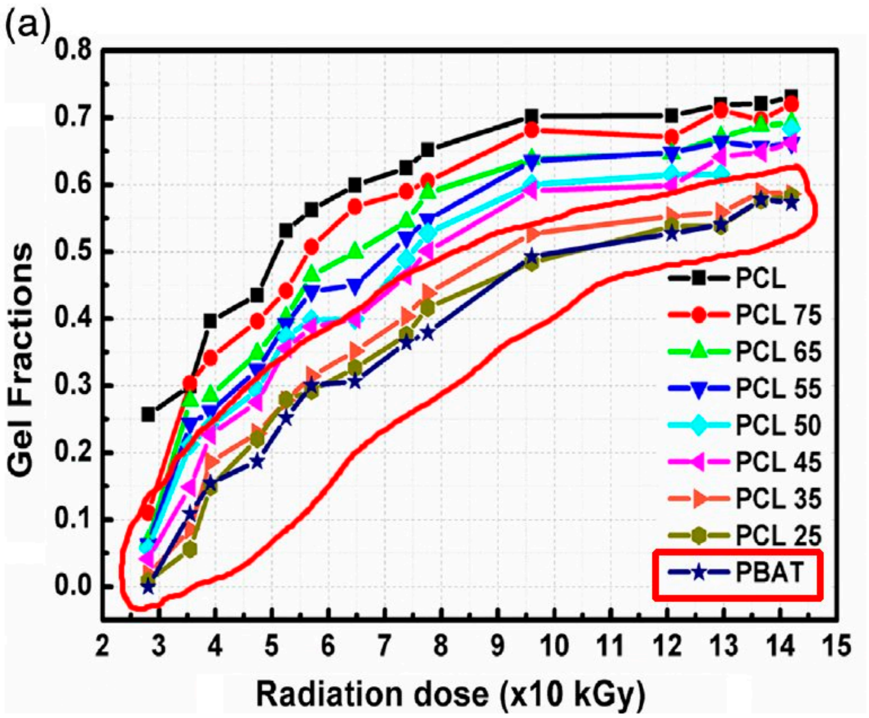

Figure S6. Gel data for irradiated PBAT with 2% weight of crosslinker TAIC (black line with stars) (taken from Reference [26])

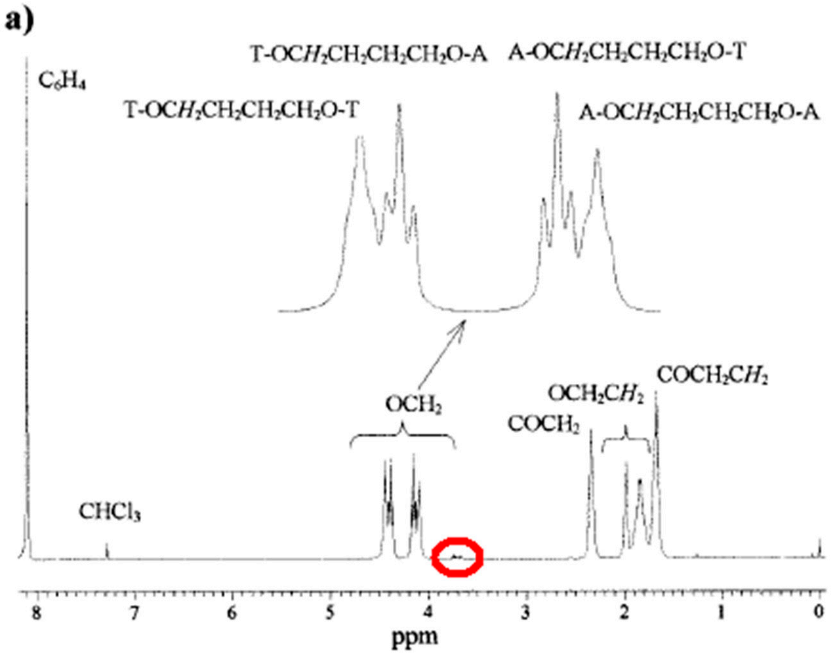

Figure S7. PBAT spectra from reference [44] with signals for terminal groups highlighted

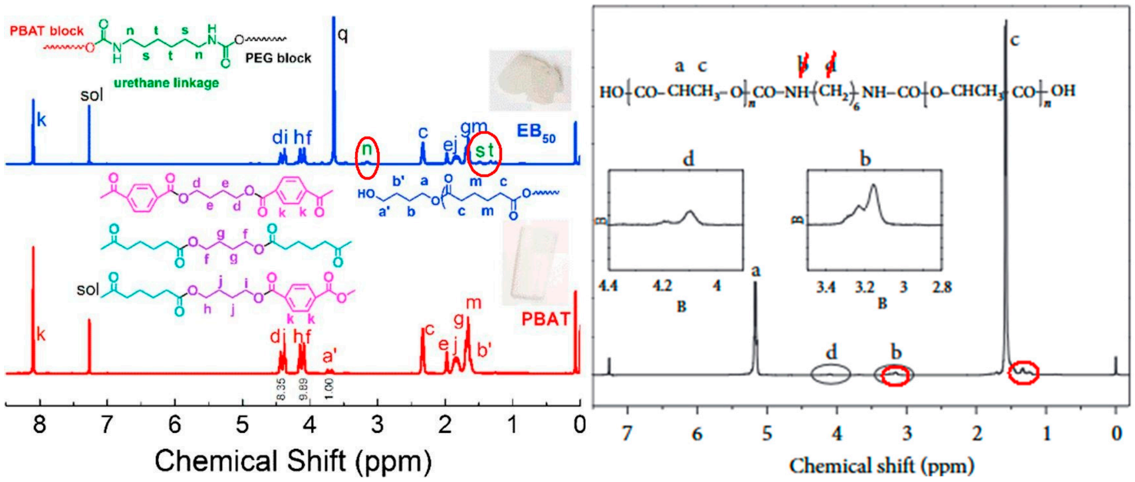

Figure S8. Proton NMR signals for the hexamethylene diurethane (inside red circles) from References [54] (left) and [55] (right): Protons are not correctly assigned in Ref. [55]

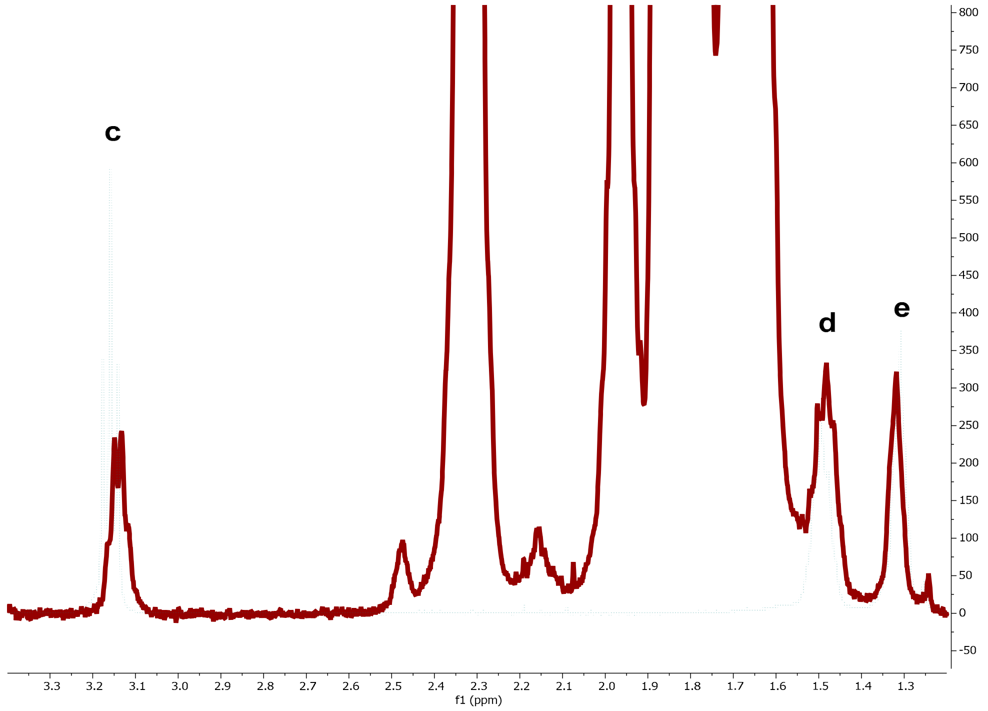

Figure S9. Proton NMR signals for unirradiated PBAT (red colour) and for a polyurethane prepared from polycarbonate diol and hexamethylene diisocyanate (blue colour)

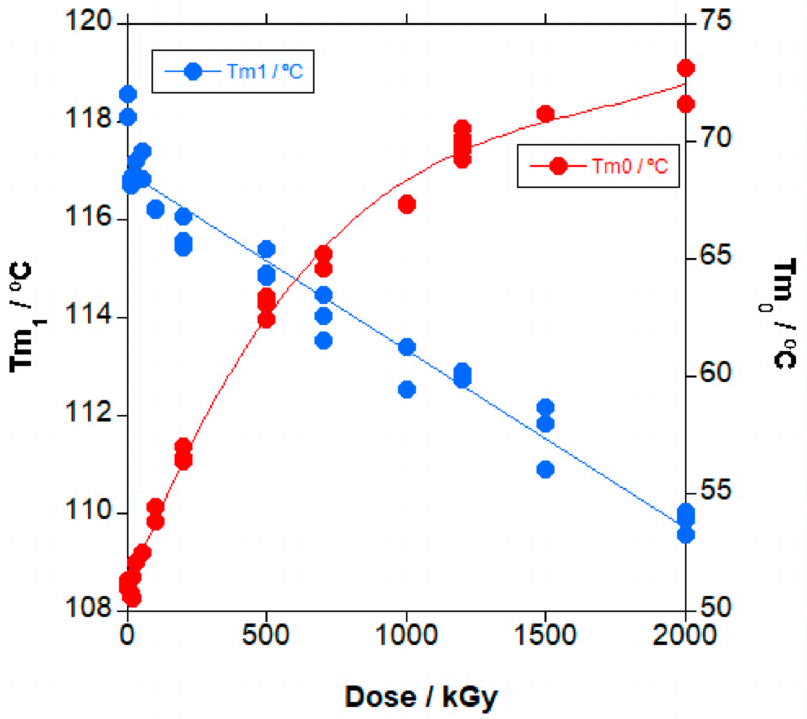

Figure S10. Main melting peak (Tm1) and secondary melting peak (Tm0) in the first heating step for irradiated PBAT vs. dose

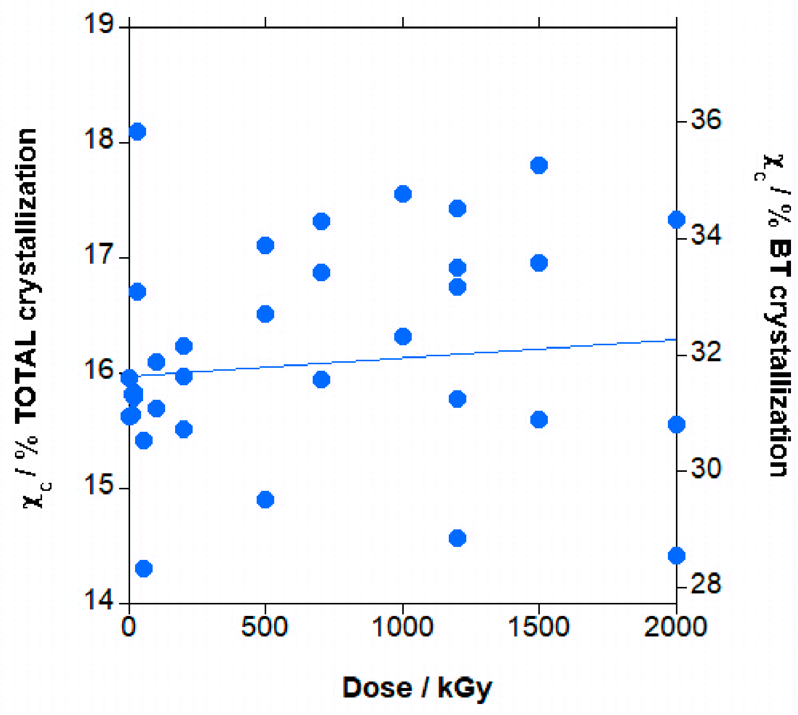

Figure S11. Crystallinity in the cooling step (χc) for irradiated PBAT vs. dose

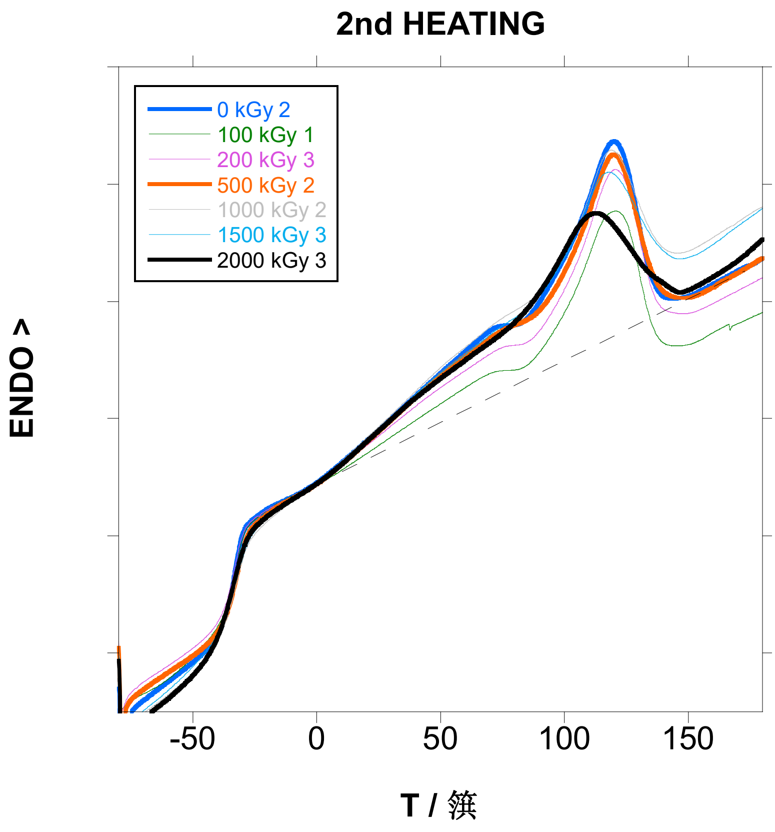

Figure S12. Second heating curves for irradiated PBAT at different doses

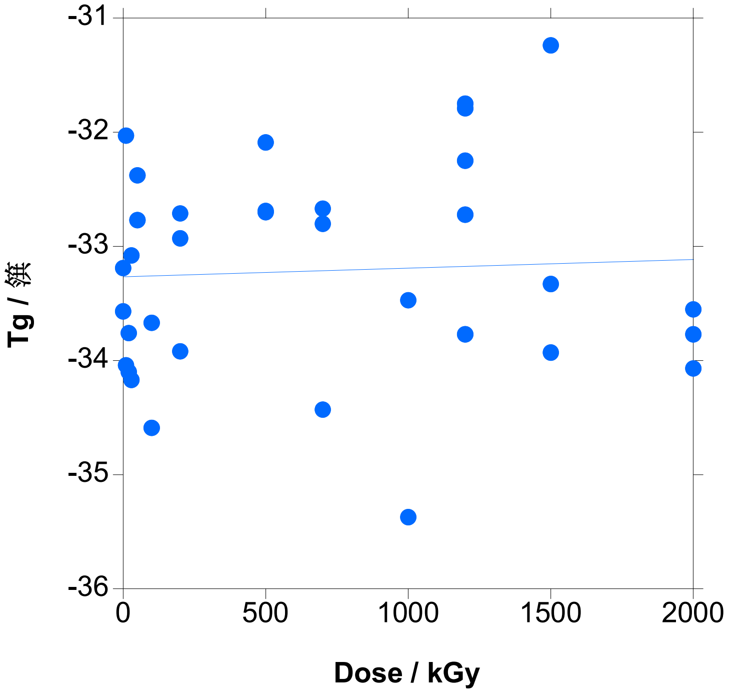

Figure S13. Tg vs. dose for irradiated PBAT at different doses (second heating step)

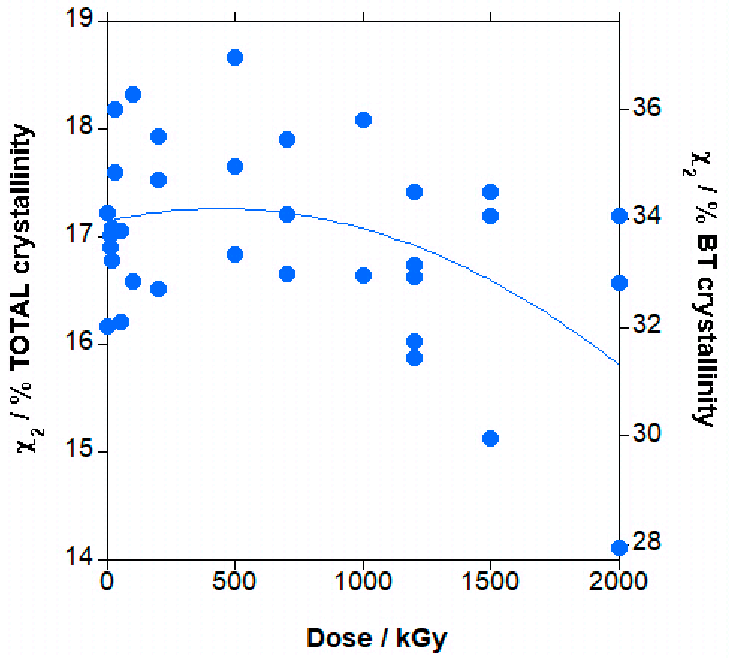

Figure S14. Crystallinity in the second heating step (χ2) for irradiated PBAT vs. dose

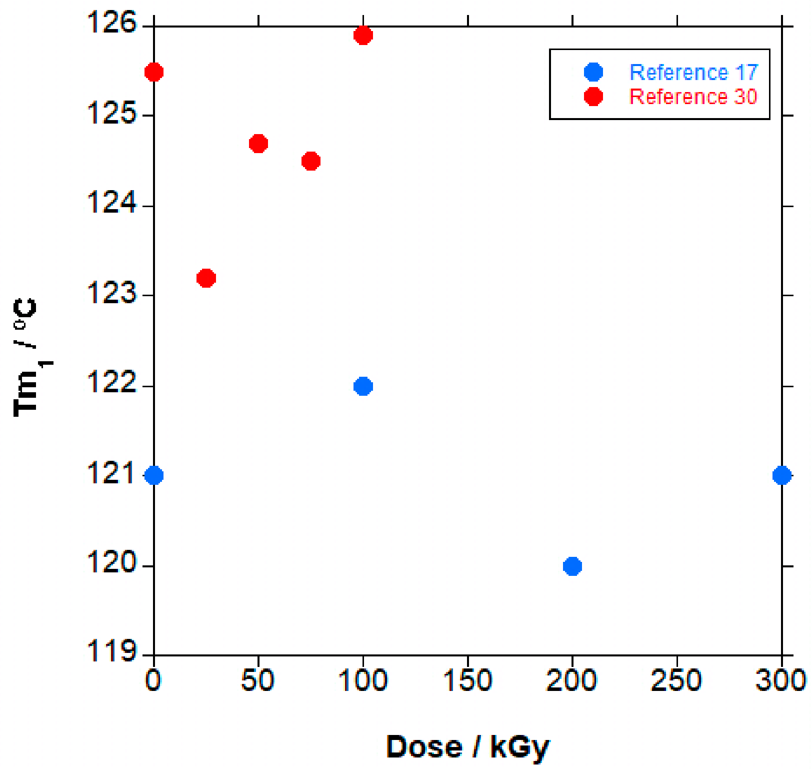

Figure S15. Melting temperature in the first heating cycle (Tm1) for irradiated PBAT (data taken from references [19,32])

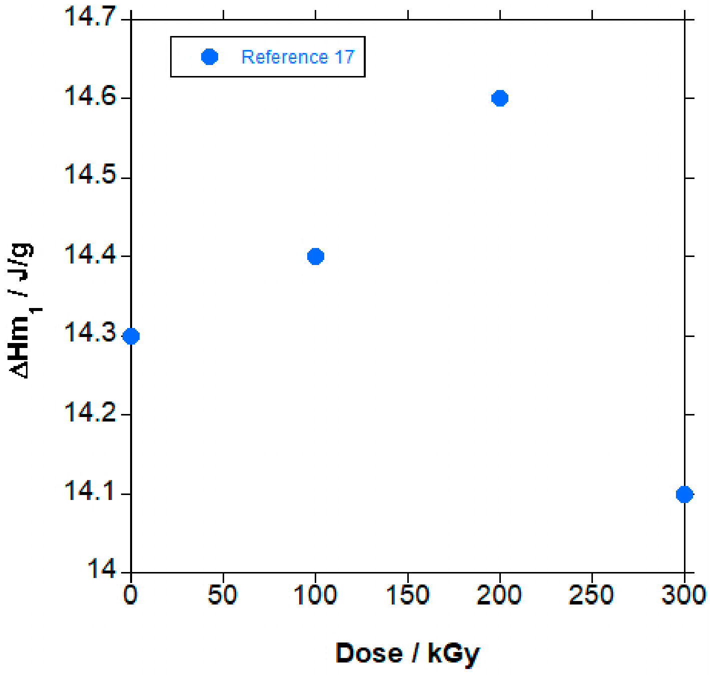

Figure S16. Enthalpy of melting in the first heating cycle for irradiated PBAT (data taken from Ref. [19])

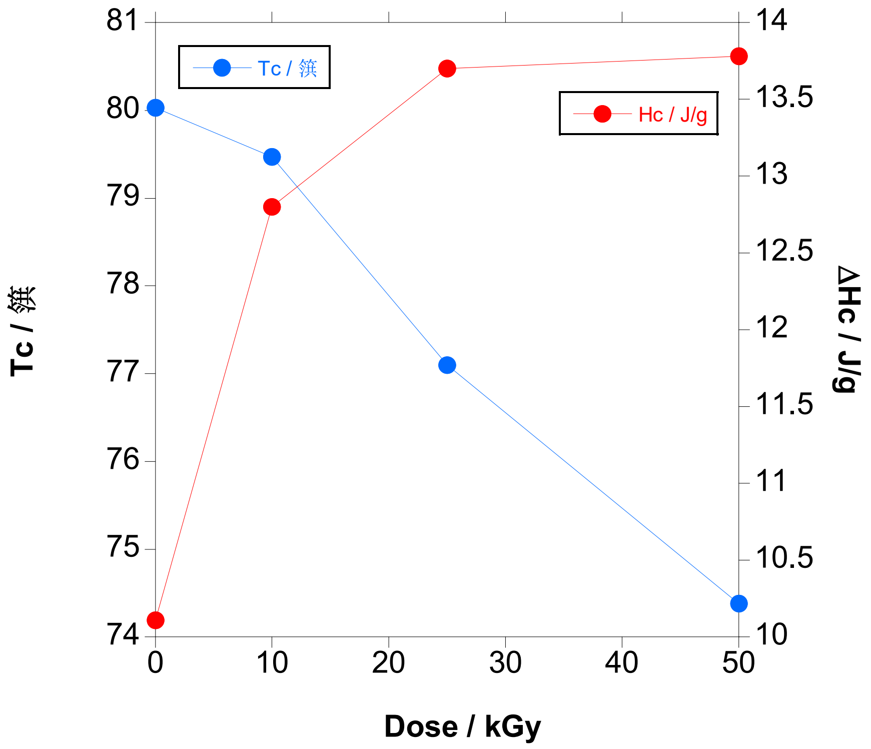

Figure S17. Temperature of crystallization (blue) and heat of crystallization (red) for irradiated PBAT (data taken from Ref. [24])

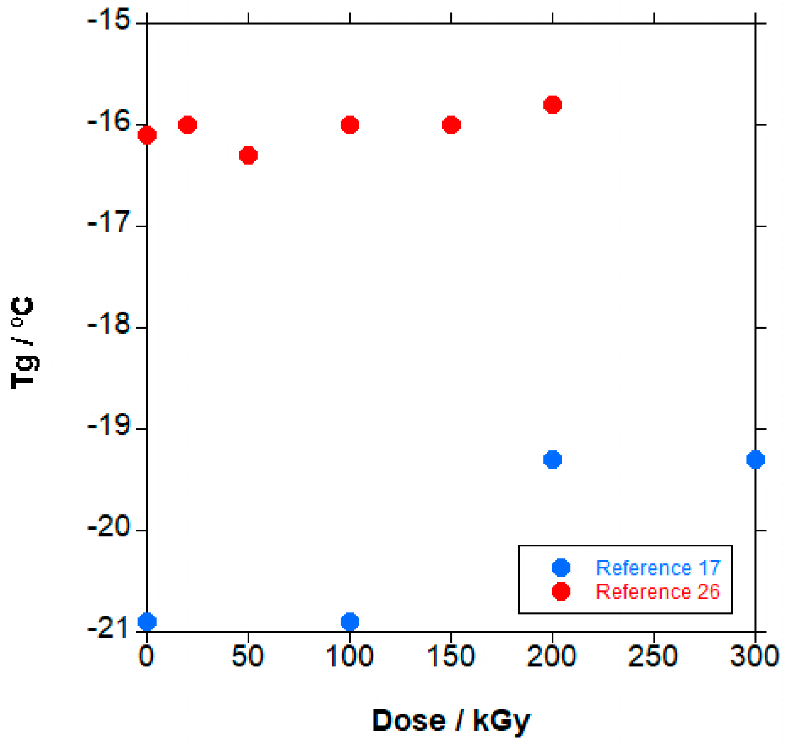

Figure S18. Glass transition temperature for irradiated PBAT by using DMTA analysis (data taken from References [19,28])

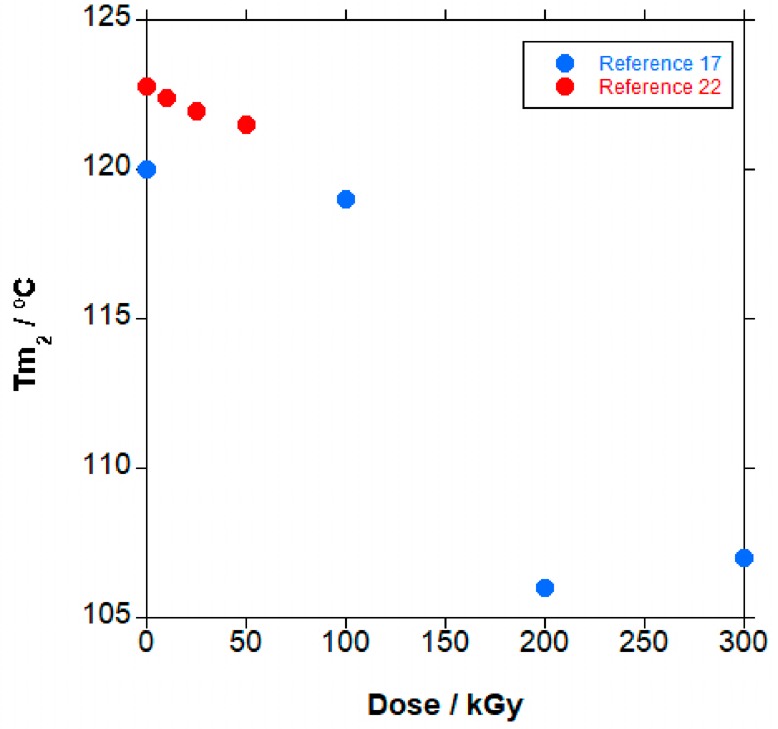

Figure S19. Temperature of melting in the second heating cycle for irradiated PBAT (data taken from References [19,24])

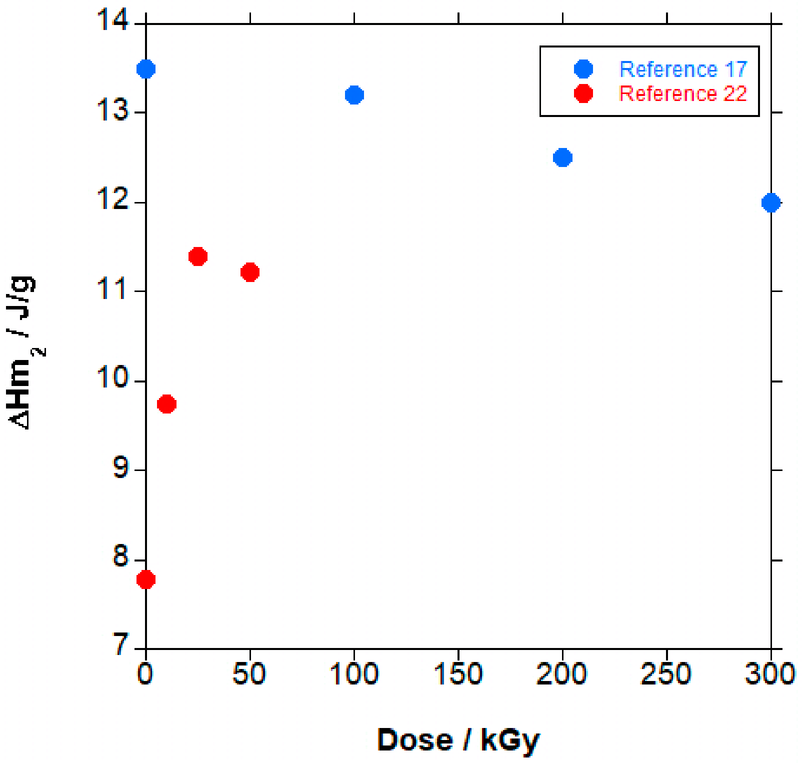

Figure S20. Melting enthalpy in the second heating cycle for irradiated PBAT (data taken from References [19,24])

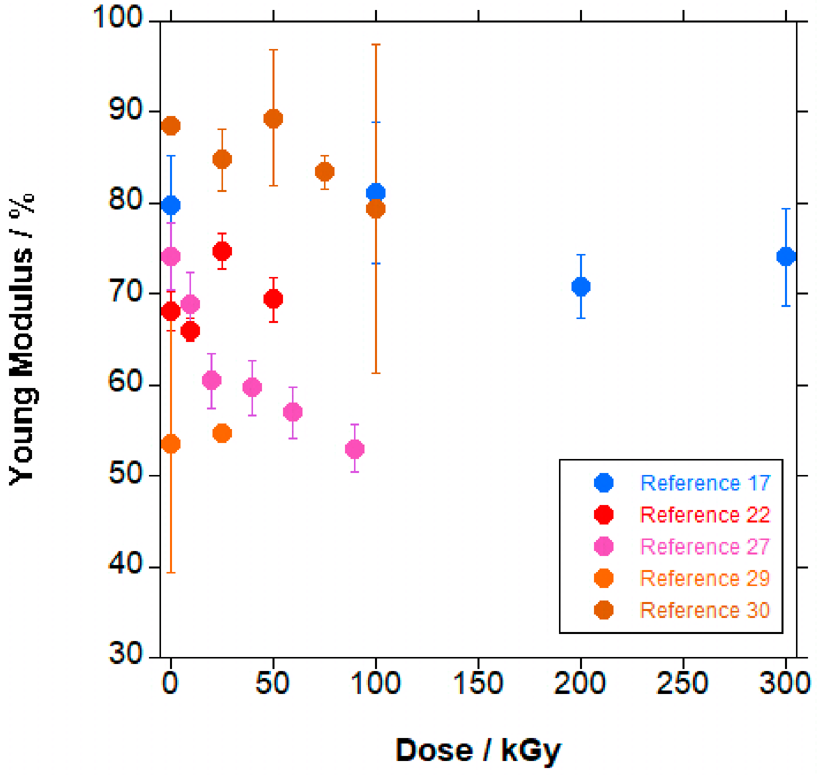

Figure S21. Young's Modulus for irradiated PBAT (data taken from References [19,24,29,31,32])

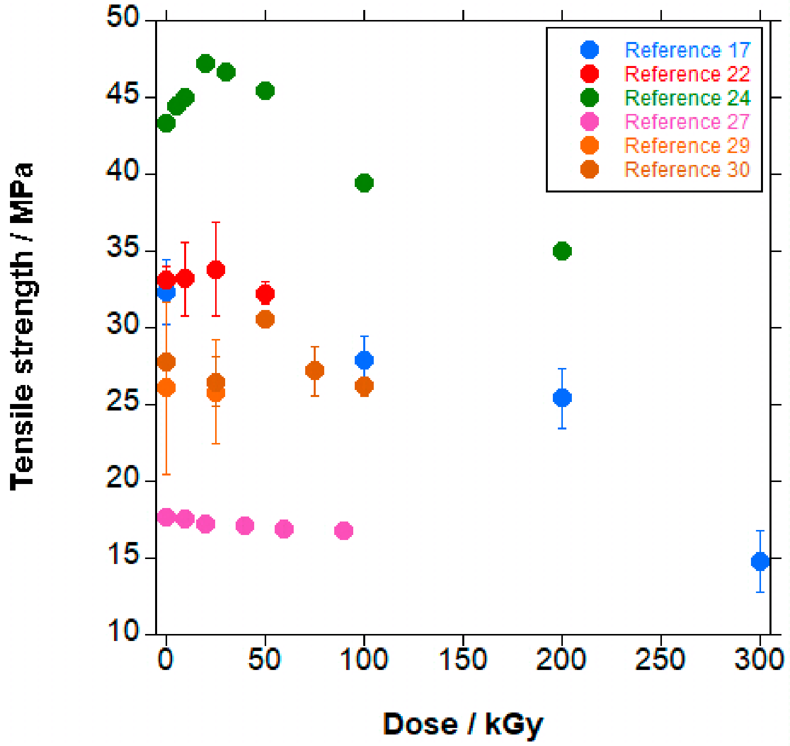

Figure S22. Tensile strength for irradiated PBAT (data taken from References [19,24,26,29,31,32])

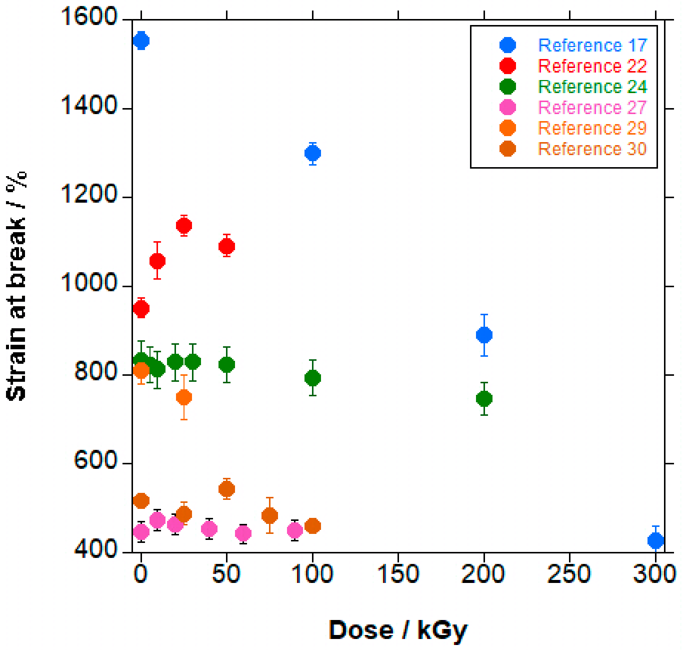

Figure S23. Strain at break for irradiated PBAT (data taken from References [19,24,26,29,31,32])

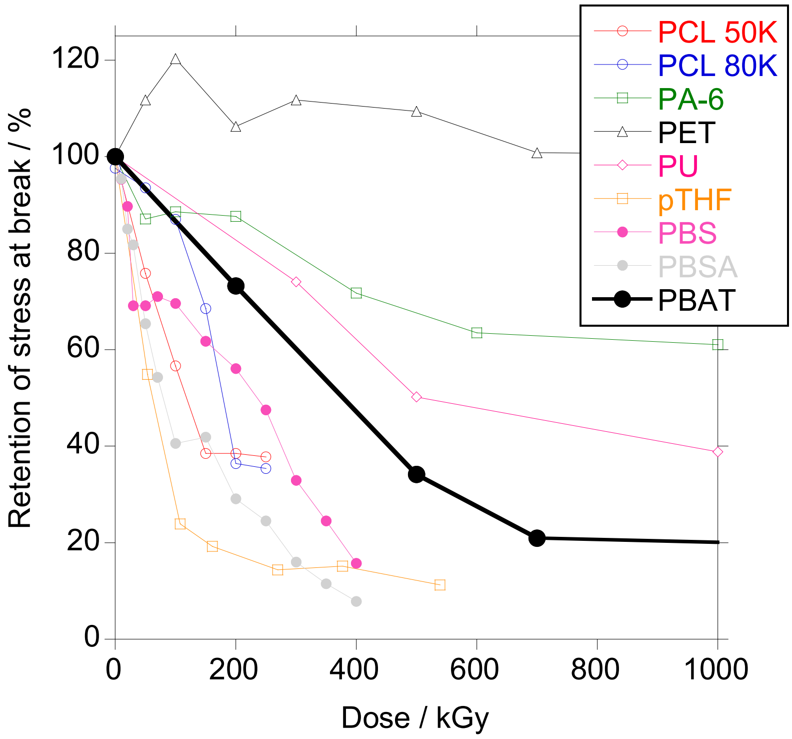

Figure S24. Retention of tensile strength vs. dose for PBAT (black thick circles), PBS (purple circles), PBSA (grey circles), PCL 50K (red circles), PCL 80K (blue circles), PA-6 (green squares), PET (black triangles), aliphatic polyurethane (magenta diamonds) and poly(tetramethylene oxide) (orange squares).
